# IFNγ Transcribed by IRF1 in CD4^+^ Effector Memory T Cells Promotes Senescence-Associated Pulmonary Fibrosis

**DOI:** 10.14336/AD.2023.0320

**Published:** 2023-12-01

**Authors:** Haiyun Chen, Qiuyi Wang, Jie Li, Yuan Li, Ao Chen, Jiawen Zhou, Jingyu Zhao, Zhiyuan Mao, Zihao Zhou, Jin’ge Zhang, Yue Wang, Rong Wang, Qing Li, Yongjie Zhang, Runqiu Jiang, Dengshun Miao, Jianliang Jin

**Affiliations:** ^1^Department of Human Anatomy, Research Centre for Bone and Stem Cells; Key Laboratory for Aging & Disease;; The State Key Laboratory of Reproductive Medicine; Nanjing Medical University, Nanjing, Jiangsu, China. ^2^Medical School of Nanjing University, Jiangsu Laboratory of Molecular Medicine, Nanjing University, Nanjing, Jiangsu, China. ^3^Department of Orthopaedics, Xuzhou Central Hospital; The Xuzhou Clinical School of Xuzhou Medical University; The Xuzhou School of Clinical Medicine of Nanjing Medical University, Xuzhou, Jiangsu, China. ^4^The Research Center for Aging, Affiliated Friendship Plastic Surgery Hospital of Nanjing Medical University, Nanjing, Jiangsu, China. ^5^Department of cardiology, The Affiliated Suzhou Hospital of Nanjing Medical University, Suzhou Municipal Hospital, Gusu School, Nanjing Medical University, Suzhou, Jiangsu, China. ^6^Department of Science and Technology, Jiangsu Jiankang Vocational College, Nanjing, China.

**Keywords:** CD4^+^ effector memory T cells, IFNγ, IRF1, epithelial-to-mesenchymal transition, alveolar type II epithelial cells, TGF-β1/IL-11/MEK/ERK signals;, senescence, pulmonary fibrosis

## Abstract

Physiologically aged lungs are prone to senescence-associated pulmonary diseases (SAPD). This study aimed to determine the mechanism and subtype of aged T cells affecting alveolar type II epithelial (AT2) cells, which promote the pathogenesis of senescence-associated pulmonary fibrosis (SAPF). Cell proportions, the relationship between SAPD and T cells, and the aging- and senescence-associated secretory phenotype (SASP) of T cells between young and aged mice were analyzed using lung single-cell transcriptomics. SAPD was monitored by markers of AT2 cells and found to be induced by T cells. Furthermore, IFNγ signaling pathways were activated and cell senescence, SASP, and T cell activation were shown in aged lungs. Physiological aging led to pulmonary dysfunction and TGF-β1/IL-11/MEK/ERK (TIME) signaling-mediated SAPF, which was induced by senescence and SASP of aged T cells. Especially, IFNγ was produced by the accumulated CD4^+^ effector memory T (T_EM_) cells in the aged lung. This study also found that physiological aging increased pulmonary CD4^+^ T_EM_ cells, IFNγ was produced mainly by CD4^+^ T_EM_ cells, and pulmonary cells had increased responsiveness to IFNγ signaling. Specific regulon activity was increased in T cell subclusters. IFNγ transcriptionally regulated by IRF1 in CD4^+^ T_EM_ cells promoted the epithelial-to-mesenchymal transition by activating TIME signaling and cell senescence of AT2 cells with aging. Accumulated IRF1^+^CD4^+^ T_EM_ produced IFNγ in lung with aging and anti-IRF1 primary antibody treatment inhibited the expression of IFNγ. Aging might drive T cell differentiation toward helper T cells with developmental trajectories and enhance cell interactions of pulmonary T cells with other surrounding cells. Thus, *IFNγ* transcribed by IRF1 in CD4^+^ effector memory T cells promotes SAPF. IFNγ produced by CD4^+^ T_EM_ cells in physiologically aged lungs could be a therapeutic target for preventing SAPF.

## INTRODUCTION

The degeneration of pulmonary structure and function and an enhanced susceptibility to pulmonary diseases are correlated with immunosenescence [1]. As an important factor leading to immunosenescence, T cell aging leads to increased susceptibility of elders to foreign pathogens [2]. Elderly persons have the highest risk of being infected with COVID-19 and account for about 80% of COVID-19-related deaths [3]. Thus, exploring the effect and contribution of aging T cells to increased susceptibility to COVID-19 infection and senescence-associated pulmonary diseases (SAPD) is urgent and necessary.

Immunity to foreign pathogens is impaired with downregulated CD4^+^ and CD8^+^ T cell responses during immunosenescence [4]. As a hallmark of T cell aging, naïve CD4^+^ and CD8^+^ T cells decline. However, the effector memory T (T_EM_) cells expand with age in mice and humans [5, 6]. Tissue-resident T_EM_ cells have been reported in both the pathogenesis of and protection from chronic inflammatory diseases [7-10]. Previous reports have demonstrated that pro-inflammatory factors, including interferon-gamma (IFNγ), perforin, and granzymes secreted by T cells, may damage stromal cells, causing lung injury and chronic inflammatory diseases [7, 11]. However, the effects of accumulated T_EM_ cells on pulmonary cells and on pathogenesis of SAPD are still unclear.

IFNγ is secreted primarily by T lymphocytes or natural killer (NK) and natural killer T (NKT) cells [12-14]. In recent years, research on interferon has focused on anti-tumor and immunomodulatory effects. However, the effects and mechanism of interferon on tissue stromal cells are insufficient, especially in elderly individuals. It has been reported that IFNγ can induce epithelial-to-mesenchymal transition (EMT) through the JAK-STAT signaling pathway in prostate cancer [15]. Reported data has associated IFNγ derived from T cells and epithelial mediators with severe asthma [16]. Mice with acute lung injury could be rescued by anti-IFNγ therapy [17]. Mounting evidence has demonstrated that IFNγ and other inflammatory factors secreted by T cells are involved in the development of chronic lung diseases [2, 16]. A previous study finds that T cells from old brains express IFNγ, and the IFNγ response induced by T cells appears to be detrimental for neural stem cell function [18]. However, with age, the subgroup of T cells producing IFNγ, the cells in the lung that are sensitive to the IFNγ response, and the role of IFNγ in the occurrence and process of SAPD remain unclear. At present, the mechanism of transcriptional modulation of IFNγ in aged lung has not been reported. Previous studies have indicated that IRF1 mediates IFN-I (IFN-α/β) and IFN-III (IFN-λ) production [19], but whether *IFNγ* is transcribed by IRF1 remains unknown.

Previous studies have revealed that aging immunity contributes to the progression of pulmonary fibrosis [1]. Pulmonary fibrosis is a primary pathological change in the process of pneumonia [20]. Several lines of evidence have suggested that human alveolar type II epithelial (AT2) cells undergo a transforming growth factor-β (TGF-β)-dependent EMT, which is important in pulmonary fibrosis, and that this is mediated by the TGF-β and ERK pathways [21]. In our recent study, TGF-β1/IL-11/MEK/ERK (TIME) signaling mediated senescence-associated pulmonary fibrosis (SAPF) by promoting EMT of AT2 cells and type-Ι-collagen production by aging pulmonary fibroblasts [22]. However, whether AT2 cells are sensitive to IFNγ and whether IFNγ could induce EMT by activating TIME signaling are unclear.

This study demonstrated that physiological aging could lead to pulmonary dysfunction, cellular senescence, and TIME signaling-mediated SAPF. This induced senescence and SASP of pulmonary T cells, especially in accumulated CD4^+^ T_EM_ cells in physiologically aged lungs. Increased secretions of IFNγ mainly transcribed by IRF1 in CD4^+^ T_EM_ cells promoted EMT of AT2 cells by activating TIME signaling. Thus, the IFNγ produced by CD4^+^ T_EM_ cells in physiologically aged lung could be a therapeutic target for preventing SAPF.

## MATERIALS AND METHODS

### Data acquisition and data processing

The mouse lung scRNA-seq data described by Angelidis et al. [23] was derived from young and physiologically aged mice. The single-cell suspension was obtained according to the method reported by Angelidis et al. [23], and the dataset was downloaded from the Gene Expression Omnibus under the accession number GSE124872.

The Seurat R package (version 4.1.0) [24] was used for quality control of single cells, data normalization, dimension reduction, and unsupervised clustering. Cells with fewer than 200 genes or more than 2500 genes or over 10% mitochondrial genes were further excluded from the downstream analyses. The ‘NormalizeData’ and ‘ScaleData’ functions were used to normalize and center the expression levels in the dataset for dimensional reduction with default parameters. Total cell clustering was performed by the ‘FindClusters’ function at a resolution of 0.5. Dimensionality reduction was performed with the ‘RunUMAP’ function and visualized by uniform manifold approximation and projection (UMAP). Differentially expressed genes (DEGs) between old and young mice were determined with the Wilcoxon rank-sum test using the ‘FindAllMarkers’ function. Those with |avg_logFC| > 0.25 and p_val_adj < 0.05 were considered as DEGs.

### Gene set score analysis

The ‘AddModuleScore’ function from the Seurat R package was used to calculate the module scores for each input cell. Different gene set scores between young and old samples were analyzed using the ggpubr package via the Wilcox test (https://rpkgs.datanovia.com/ggpubr/index.html) (version 0.4.0). Genesets were obtained from the MSigDB database (www.gsea-msigdb.org/gsea/msigdb/) [25].

We used the ‘AddModuleScore’ function to calculate the average expression levels of each cluster on the single cell level, subtracted by the aggregated expression of the control gene sets. All analyzed genes were binned based on the averaged expression. Briefly, the average expression level of each gene in the gene set was calculated in every cell. The complete list of SASP genes used is shown in the Supplementary Table 1.

### Pseudotime analysis

T cell trajectory inference was determined using the Monocle2 R package with the default settings given there (http://cole-trapnell-lab.github.io/monocle-release/) [26]. The significant genes defining the progress of the trajectory were obtained using the function differential GeneTest (fullModelFormulaStr = ~clusters). Pseudotime ordering was performed using the function ‘reduce dimension’ with the reduction_method set as the DDRTree. Based on the pseudotime analysis, branch expression analysis modeling (BEAM analysis) was applied for branch fate determined gene analysis as previously described [26].

Columns are points in pseudotime, rows are genes, and the beginning of pseudotime is in the middle of the heatmap. Reading from the middle of the heatmap to the right, one lineage can be followed through pseudotime. Reading to the left, other lineages can be followed. The genes are clustered hierarchically. Therefore, modules of genes with similar lineage-dependent expression patternscan be visualized, as previously described [26].

### Cell-cell communication analysis

To assess cell-cell communication molecules between different cell types, the CellChat R package (version 1.1.3) was used [27] (https://github.com/sqjin/CellChat) to infer the intercellular communication network from single-cell transcriptome data. Young and aged mouse single-cell data were integrated into CellChat object, and the ‘computeCommunProbPathway’ function was used to compute the communication probability at the signaling pathway level by summarizing all related ligands/ receptors with type = "truncatedMean" and trim = 0.01 parameters. The contribution of each ligand-receptor pair from T cells to other cell types was computed and visualized using the ‘netVisual_bubble’ function. The ‘getMaxWeight’ and ‘netVisual_aggregate’ functions were used to calculate and visualize IFNγ signaling from T cells to other cell types.

### Transcription factor activity analysis

To assess transcription factor regulation activity, the R package SCENIC (version 1.2.4) was used for inference activity of transcription factors and their target genes (regulons). SCENIC is a computational workflow that predicts transcription factor (TF) activities from scRNA-seq data [28] (https://scenic.aertslab.org/). Regulons in individual cells were scored by AUCell with the ‘runSCENIC_3_scoreCells’ function.

### Gene enrichment analysis

To characterize the biological characteristics of a given gene set, such as pathway activation, ‘ClusterProfiler’ R package (version 4.2.2) was applied for gene enrichment analysis [29]. DEGs were used for kyoto encyclopedia of genes and genomes (KEGG), gene ontology (GO), and disease enrichment analysis according to the developer’s manual (https://yulab-smu.top/biomedical-knowledge-mining-book/index.html).

### Age-associated pathway enrichment analysis

The method used by Dulken et al. [18] was used for the GSEA algorithm to investigate the broad signature of aging in each lung cell type sequenced. For each cell type, genes were ranked by decreasing MAST-derived Z-scores with positive Z-scores corresponding to enrichment in older cells. See https://github.com/gitbuckley/SingleCellAgingSVZ for specific codes.

### Mice

Two-year-old wild type (WT) and 2-month-old WT mice with a C57BL/6J background were purchased from the Laboratory Animal Research of Nanjing Medical University in Nanjing, China. All the animals in this study were fed a normal diet, which contained 1.0% calcium, 0.67% phosphorus, and 2.2-IU vitamin D/g (#1010013; Jiangsu Province Collaborative Medicine Bioengineering Co., Ltd., Nanjing, China). This study was performed in strict accordance with the guidelines of the Institute for Laboratory Animal Research of Nanjing Medical University in Nanjing, China. The protocol was approved by the Committee on the Ethics of Animal Experiments of Nanjing Medical University (Permit Number: IACUC-1802007).

### Cell cultures

*AT2 cells:* AT2 cells were separated from 2-month-old WT mice, cultured, and detected by Western blotting for the marker surfactant protein C (SFTPC), as previously described [30-32].

*HEK293T cell line:* Human HEK293T cells were cultured in 90% DMEM (ZQ100, Zhong Qiao Xin Zhou Biotechnology, China) with 10% FBS (ZQ500-S, Zhong Qiao Xin Zhou Biotechnology, China), 100 U/mL penicillin, and 0.1 mg/mL streptomycin (CSP006, Zhong Qiao Xin Zhou Biotechnology, China).

*Pulmonary CD4^+^ effector memory T cells:* Mouse lungs were digested with 1 mg/mL collagenase to prepare a single-cell suspension. Pulmonary CD4^+^ effector memory T cells were sorted with a BD Aria II and cultured in 90% 1640 medium (ZQ200, Zhong Qiao Xin Zhou Biotechnology, China) with 10% FBS (ZQ500-S, Zhong Qiao Xin Zhou Biotechnology, China), 100 U/mL penicillin, and 0.1 mg/mL streptomycin (CSP006, Zhong Qiao Xin Zhou Biotechnology, China).

### Plasmid construction and transfection

The *Irf1* gene was cloned into the pcDNA3.1 vector (TranSheep Bio Co., Ltd., Shanghai, China). Lipofect-amine® 2000 reagent (Invitrogen, USA) was used to transfect plasmid into Human HEK293T cells following the manufacturer's instructions.

### Administration of drugs or reagents

*Recombinant mouse IFNγ, anti-IFNγ* primary antibody, *and anti-IRF1* primary antibody*:* AT2 cells were treated for 48 h with different concentrations (0, 20, 40, 60, 80, and 100 ng/mL) of mouse IFNγ (Chamot Biotechnology Inc., Shanghai, China), sorted cells, and anti-IFNγ primary antibody (#15365, Proteintech, USA).

Pulmonary CD4^+^ effector memory T cells were treated for 48 h with anti-IRF1 primary antibody (#11335, Proteintech, USA).

### Pulmonary function analysis

Mice were placed in the plethysmography chamber for a whole-body plethysmograph (WBP-8MR, TOW-INT TECH, Shanghai, China). After 15 min acclimation in the chamber, the unrestrained mice were monitored for 15 minutes. The inspiration time, expiration time, peak inspiratory flow, tidal volume, minute volume, accumulated volume, expiratory flow 50 (expiratory flow at 50% volume), relaxation time, end-expiratory pause, enhanced pause, and ratio of expiration time were determined every 60 seconds by the software (ResMass 1.4.2.8, TOW-INT TECH, China) as previously described [33-36]. The above indicators were officially recorded after 5 consecutive days of testing.

### Preparation of pulmonary sections

Pulmonary samples from mice anesthetized and perfused as previously described were cut into small pieces and postfixed in periodate-lysine-paraformaldehyde (PLP) solution for 24 hours at 4°C, as previously described [37]. For histochemistry or immunohistochemistry, sections were dehydrated in a series of graded ethanol solutions, embedded in paraffin, and cut into 5-μm sections using a rotary microtome (Leica Biosystems Nussloch GmbH, Nussloch, Germany), as previously described [38].

The samples were embedded in Optimal Cutting Temperature (O.C.T.) compound (#4583, SAKURA Finetek USA, Inc., CA, USA) and sliced into 7-μm thick sections with a freezing microtome (Thermo Scientific Cryotome FSE Cryostats, Loughborough, Leicestershire). The sections were used for immunofluorescence staining.

### Immunofluorescent staining of pulmonary sections

Primary antibodies against IRF1 (#11335, Proteintech, USA) and CD4 (#100507, BioLegend, USA), fluorophore-conjugated antibodies for CD4-PE (#12-0042-82, eBioscience, USA) and CD44-PE-Cy5 (#15-0441-81, eBioscience, USA), and IFNγ-FITC (#505806, BioLegend, USA) were used. Dylight594-conjugated secondary antibody (goat anti-rabbit IgG, BS10029, Bioworld Technology, USA) and Dylight649-conjugated secondary antibody (goat anti-rabbit IgG, BS60034, Bioworld Technology, USA; goat anti-mouse IgG, GAM6492, Multi Sciences Biotech, Co., Ltd., China) were used. Secondary antibody only controls were used in all immunofluorescence staining experiments.

### Histology staining

*Pre-embedding Senescence-Associated β-galactosidase (SA-β-gal) staining:* Pulmonary samples from mice were stained following previously described protocols [38-40].

*SA-β-gal staining:* SA-β-gal staining was performed with cryosections of pulmonary tissue using the senescence β-galactosidase staining kit (#C0602, Beyotime Institute of Biotechnology, Shanghai, China) according to the manufacturer’s instructions and as previously described [41]. Serial paraffin sections were deparaffinized and rehydrated for histochemical or immunohistochemical staining.

*Masson’s trichrome staining:* Serial paraffin sections were stained with Masson’s detection kits (#D026, Nanjing Jiancheng Bioengineering Institute, Nanjing, Jiangsu, China) according to the manufacturer’s instructions and as previously described [38, 39].

*Immunohistochemical staining:* Staining was performed as previously described. Primary antibodies against p16 (ab211542, Abcam, Cambridge, MA, USA), p53 (sc-126, Santa Cruz Biotechnology Inc., Dallas, TX, USA), alpha smooth muscle actin (α-SMA) (ab28052, Abcam, USA), collagen 1 (#1310-08, Southern Biotech, Birmingham, AL, USA), TGF-β1 (ab64715, Abcam, USA), Interleukin-11 (IL)-11 (sc-133063, Santa Cruz Biotechnology Inc., USA), IL-11Ra1 (sc-130920, Santa Cruz Biotechnology Inc., USA), CD3e (sc-20047, Santa Cruz Biotechnology Inc., USA), IL-17A (sc-374218, Santa Cruz Biotechnology Inc., USA), and IFNγ (#15365, Proteintech, USA) were used. After washing, the sections were incubated with secondary antibody (biotinylated IgG; Sigma-Aldrich), washed, and processed using Vectastain ABC-HRP kits (Vector Laboratories Inc., Burlingame, CA, USA). Secondary antibody-only controls were acquired in all immunohistochemical staining experiments.

### Western blots

Western blots were generated as previously described. Primary antibodies against p19 (ab80, Abcam, USA), p21 (sc-471, Santa Cruz Biotechnology Inc., USA), p16 (ab211542, Abcam, USA), p53 (sc-126, Santa Cruz Biotechnology Inc., USA), SFTPC (ab211326, Abcam, USA), collagen 1 (#1310-08, Southern Biotech), α-SMA (ab5694, Abcam, USA), TGF-β1 (ab64715, Abcam, USA), Smad2 (sc-101153, Santa Cruz Biotechnology Inc., USA), phospho-Smad2 (Ser465/467) (#3108, Cell Signaling Technology, USA), Snail (#3879, Cell Signaling Technology, USA), IL-11 (sc-133063, Santa Cruz Biotechnology Inc., USA), IL-11Rα1 (sc-130920, Santa Cruz Biotechnology Inc., USA), MEK1/2 (sc-81504, Santa Cruz Biotechnology Inc., USA), phospho-MEK1/2 (sc-81503, Santa Cruz Biotechnology Inc., USA), ERK1/2 (#4695, Cell Signaling Technology, USA), pERK1/2 (Thr202/Tyr204) (#4370, Cell Signaling Technology, USA), phospho-Smad2/3 (Ser423/425) (sc-11769, Santa Cruz Biotechnology Inc., USA; #ab52903, abcam, USA), IRF1 (#11335-AP, Proteintech, USA), FLI1 (ab133485, Abcam, USA), IL-17A (sc-374218, Santa Cruz Biotechnology Inc., USA), and IFNγ (#15365, Proteintech, USA) were used. β-actin (BS6007M, Bioworld Technology, USA) was the loading control for the cytoplasmic fraction and total cell protein.

### RNA extraction and real-time RT-PCR

RNA was extracted from the spleens and mediastinal lymph nodes of mice using TRIzol reagent (#15596, Invitrogen Inc.) according to the manufacturer’s protocol. The mRNA levels in samples were quantified by real-time RT-PCR, as previously described [38, 39]. The primers are shown in the Supplementary Table 2.

### Flow cytometry and cell sorting

The lungs of mice were digested with 1 mg/mL collagenase to prepare single-cell suspensions. Lymphocytes were washed with FACS buffer (2% FBS in PBS) and stained with diluted fluorophore-conjugated antibodies for CD3-PE-Cy7 (#25-0031-82, eBioscience, USA), CD4-PE (#12-0042-82, eBioscience, USA), CD8-APC (#17-0081-82, eBioscience, USA), CD62L-FITC (#11-0621-81, eBioscience, USA), and CD44-PE-Cy5 (#15-0441-81, eBioscience, USA). Intracellular staining with antibodies for IL-17A-FITC (#506908, BioLegend, USA) and IFNγ-FITC (#505806, BioLegend, USA) was performed after stimulation with cell stimulation cocktail (#00-4975-93, eBioscience, USA). The Foxp3/ Transcription factor staining buffer (#00-5523, eBioscience, USA) was used for fixation and permeabilization as previously described [42, 43]. Single color controls were acquired in all flow cytometry experiments. Data were acquired with a FACSCalibur (BD) and analyzed with FlowJo software (Tree Star).

The spleens and lungs of mice were used to prepare single-cell suspensions. Lymphocytes were stained in FACS buffer (2% FBS in PBS) with diluted fluorophore-conjugated antibodies for CD3-PE-Cy7 (#25-0031-82, eBioscience, USA), CD4-PE (#12-0042-82, eBioscience, USA), CD8-APC (#17-0081-82, eBioscience, USA), CD62L-FITC (#11-0621-81, eBioscience, USA), and CD44-PE-Cy5 (#15-0441-81, eBioscience, USA). Cell sorting was performed with a BD Aria II as previously described [43]. Sorting efficiency was tested by FlowJo software (Tree Star).

### Chromatin immunoprecipitation

Chromatin immunoprecipitation (ChIP) was performed using the Magna ChIP™ Chromatin Immunoprecipitation A kit (Millipore, Billerica, MA, USA; 2931149) with human HEK293T cells and following the manufacturer's instructions. Antibodies against IRF1 (#11335-AP, Proteintech, USA) and rabbit IgG (#PP64, Millipore, USA) were used to incubate chromatin samples. The primers for different regions of the *IFNγ* promoter were used for analyzing the binding sites and are listed in the Supplementary Table 3.

**Figure 1.**
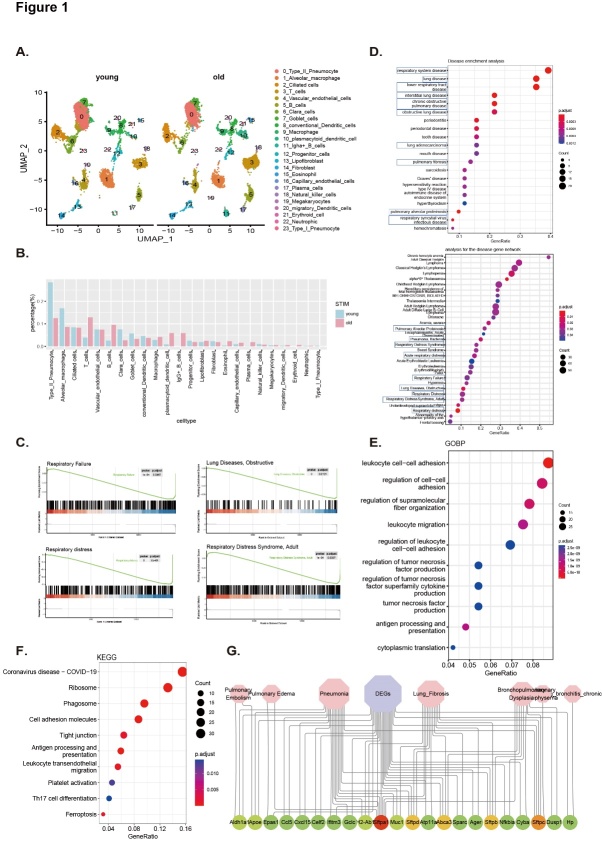
Senescence-associated pulmonary diseases are mediated by T cells. (A) Uniform manifold approximation projection (UMAP) plot of the 23 main cell types identified in young and aged lungs. (B) Percentage of different cells in total cells. (C) Genes negatively associated with respiratory failure, obstructive pulmonary disease, respiratory distress, and respiratory distress syndrome are downregulated in aged lung tissue, as identified by gene set enrichment analysis (GSEA). (D) Disease enrichment analysis. (E-F) DEGs were used for GO and KEGG analysis. (G) Network plot showing the DEGs overlapping with the lung disease database (www.malacards.org/ and www.disgenet.org/home/).

**Figure 2.**
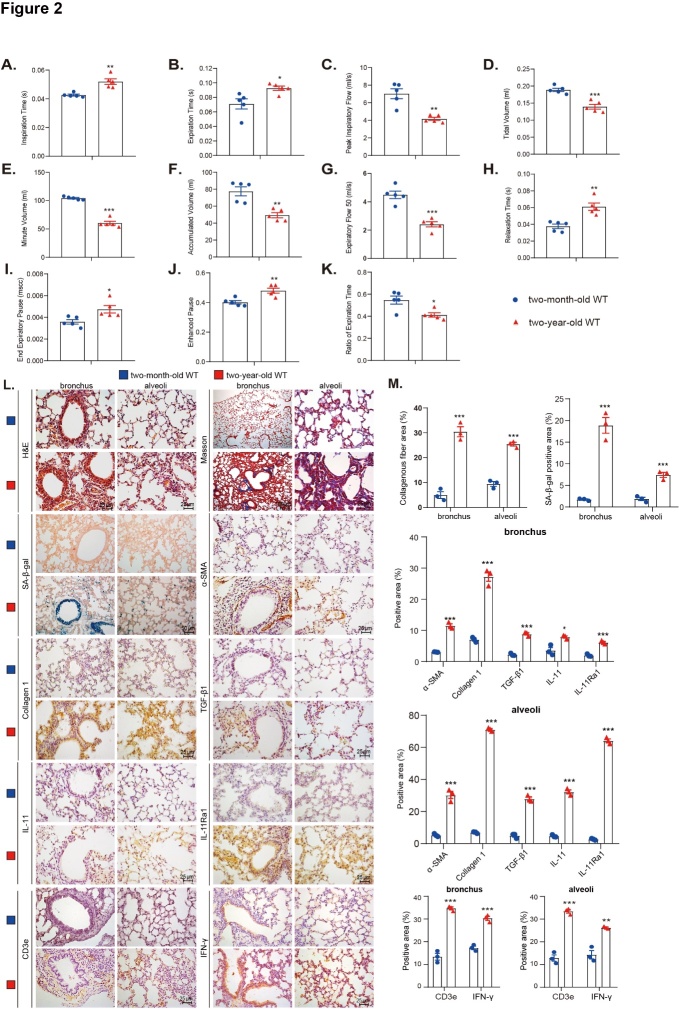
Physiological aging leads to pulmonary dysfunction and SAPF. Pulmonary function of 2-month-old and 2-year-old mice were detected by the Buxco measurement system and FinePointe analysis for (A) inspiration time (s), (B) expiration time (s), (C) peak inspiratory flow (m/s), (D) tidal volume (mL), (E) minute volume (mL), (F) accumulated volume (mL), (G) expiratory flow 50 (mL/s), (H) relaxation time (s), (I) end-expiratory pause (mscc), (J) enhanced pause, and (K) ratio of expiration time. Five mice per group were used for experiments (N = 5). Values are the mean ± SEM of five determinations per group. *p < 0.05, **p < 0.01, ***p < 0.001 compared with the 2-month-old group. Statistical analysis was performed with Student’s t-test. (L) Representative micrographs of paraffin-embedded pulmonary sections stained with hematoxylin-eosin (H&E) and Masson’s trichrome (Masson). Bronchi and alveoli sections were immunohistochemically stained for senescence-associated-β-galactosidase (SA-β-gal), α-smooth muscle actin (α-SMA), type Ι collagen (collagen 1), TGF-β1, IL-11, IL-11 receptor α1 (Rα1), CD3e, and IFNγ. Hematoxylin stained the nuclei. (M) Percentage of bronchial and alveolar areas or cells positive for Masson, SA-β-gal, α-SMA, collagen 1, TGF-β1, IL-11, IL-11Rα1, CD3e, or IFNγ. Three mice per group (N = 3) were used for experiments. Values are the mean ± SEM of three determinations per group. *p < 0.05, **p < 0.01, ***p < 0.001 compared with the 2-month-old group. Statistical analysis was performed with Student’s t-test.

### Dual-luciferase assay

The chimeric genes of the *IFNγ* promoter plasmids for transfection experiments were constructed in a pGL4.1-basic vector (TranSheep Bio Co., Ltd., Shanghai, China) by ligating the *luciferase* gene at the 5′-flanking regions of the gene upstream. HEK293T cells were plated in 24-well cell culture plates 24-hours before transfection. The mixtures of 1-μg each of pCDNA3.1-basic and pGL4.1-basic, *IRF1*-overexpression pcDNA3.1 and pGL4.1-basic, *IRF1*-overexpression pcDNA3.1 and pGL4.1-deletion (deleting the binding sequence “TATTTCTTTC CTTTTTTCTTTTTTTCTTTTTTTTTTTTTGA”), were respectively cotransfected with Firefly luciferase (Fluc)-Renilla luciferase (Rluc) into mouse HEK293T cells with the Lipofectamine® 2000 reagent (Invitrogen, USA) following the manufacturer’s instructions. Two days later, a commercial kit (Promega Corporation, Madison, WI, USA) was used to measure the promoter-driven luciferase activity.

### Statistical analysis

GraphPad Prism software (version 6.07; GraphPad Software Inc., San Diego, CA, USA) was used to analyze the data. Measurement data are described as the mean ± SD or mean ± SEM fold-change over the vehicle group. Normality of the distribution of the data was examined by the Shapiro-Wilk and the Kolmogorov-Smirnov tests. Differences in the distribution of parametric data were tested by one-way ANOVA and Student’s *t*-test, as appropriate, followed by Tukey’s post-hoc tests for multiple comparisons. One-way ANOVA is used to compare the mean value of the tested variables at three or more groups [44]. If the data are not normal or N < 6, the non-parametric alternative was used as previously described methods [45, 46]. Qualitative data are described as percentages and were analyzed using chi-square tests as indicated. P-values were two-sided and a p-value less than 0.05 was considered statistically significant.

## RESULTS

### Senescence-associated pulmonary diseases are mediated by T cells

To investigate the relationship between pulmonary immunosenescence and SAPD and its possible mechanisms, the dataset GSE124872 was re-analyzed. This dataset charts the lung single-cell atlas of young and old mice through single-cell transcriptomics. Unsupervised clustering using the Seurat package was performed to identify 23 clusters (Fig. 1A). The clusters were defined according to marker genes (Supplementary Fig. 1A). This study sought to identify senescence-associated transcriptional changes in individual pulmonary cells to elucidate the molecular mechanisms associated with aging at a cellular level. A total of 377 DEGs (|logFoldChange(logFC)| > 0.25, adjusted p-value <0.05) were differentially expressed in aged pulmonary cells compared to their younger counterparts (Supplementary Table 1). Globally, an increased proportion of many immune cell types resided in the aged pulmonary tissues. These included T cells, B cells, macrophages, eosinophils, and erythroid cells (Fig. 1B and Supplementary Fig. 1B), likely contributing to increased senescence-associated inflammation, chronic obstructive pulmonary disease (COPD), and idiopathic pulmonary fibrosis [47]. The older lungs were also accompanied by decreased AT2 cells and alveolar macrophages (Fig. 1B and Supplementary Fig. 1B).

Next, all the genes expressed between the young and old lungs were sorted according to the value of logFC to perform an unbiased GSEA analysis by the ClusterProfiler R package. Disease enrichment analysis showed a significant enrichment in respiratory failure, pulmonary obstructive disease, respiratory distress, and respiratory distress syndrome in aged lungs compared to young lungs (Fig. 1C). DEGs were used for disease over-representation analysis and showed respiratory tract disease, interstitial lung disease, obstructive lung disease, lung adenocarcinoma, pulmonary fibrosis, pulmonary alveolar proteinosis, pulmonary alveolar proteinosis, and bacterial pneumonia (Fig. 1D). GO biological process and KEGG enrichment analysis showed that DEGs were enriched mainly in antigen processing and presentation, leukocyte adhesion and migration, and T cell differentiation, indicating immune cell abnormalities during lung aging (Fig. 1E-F). Aged lungs are susceptible to coronavirus (COVID19) infection and exhibit an abnormal expression of tight intercellular junctions and cell adhesion molecules. This suggests that aging might accelerate epithelial cell shedding (Fig. 1F). The set of identified DEGs was next evaluated in databases comprising hotspot genes known to be involved in aging and various lung diseases, such as COPD, pneumonia, pulmonary fibrosis, and asthma. An overlap of the DEGs with genes in the database indicated that aging is a major contributing factor for chronic respiratory diseases and infection. The high frequency of *sftpa1*, *sftpb*, *sftpc*, and *sftpd*, which are markers of alveolar epithelial cells, suggests that these markers of alveolar epithelial cells could be used for monitoring the occurrence and development of SAPD (Fig. 1G).

**Figure 3.**
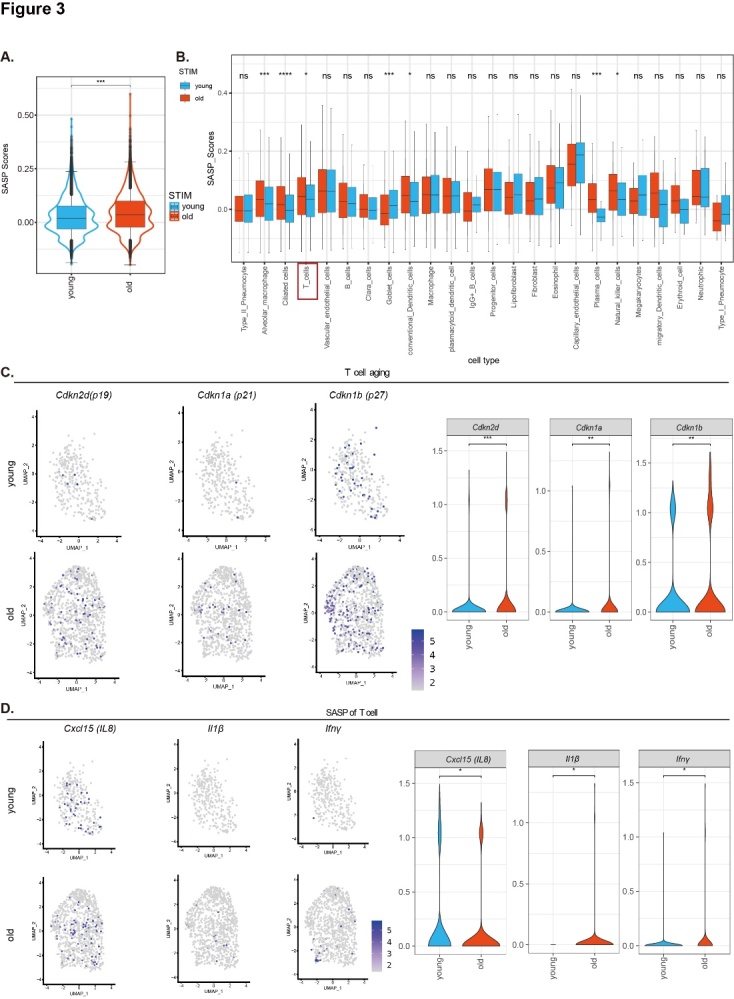
Senescence and SASP of T cells in physiologically aged lungs. (A) Gene set score analysis of SASP of different groups in the total lung cells and (B) in the various lung cells. Expression values are represented as normalized log_2_-transformed counts. Values are the mean ± SD. *p < 0.05, ***p < 0.001, ****p < 0.0001 compared with the 2-month-old WT group; ns, not significant (two-sided Wilcoxon rank-sum tests). (C) Feature plots and quantitative analysis of the average expression of for marker genes of aging (*Cdkn2d, Cdkn1a*, and *Cdkn1b*) in T cells. (D) Feature plots and quantitative analysis for marker genes of SASP (*Cxcl15(IL8), Il1β*, and *Ifnγ*) in T cells. Values are the mean ± SD and are represented as normalized log10 (value + 0.1)-transformed counts. *p < 0.05, **p < 0.01, ***p < 0.001 compared with the young group; ns, not significant (two-sided Wilcoxon rank-sum tests).

**Figure 4.**
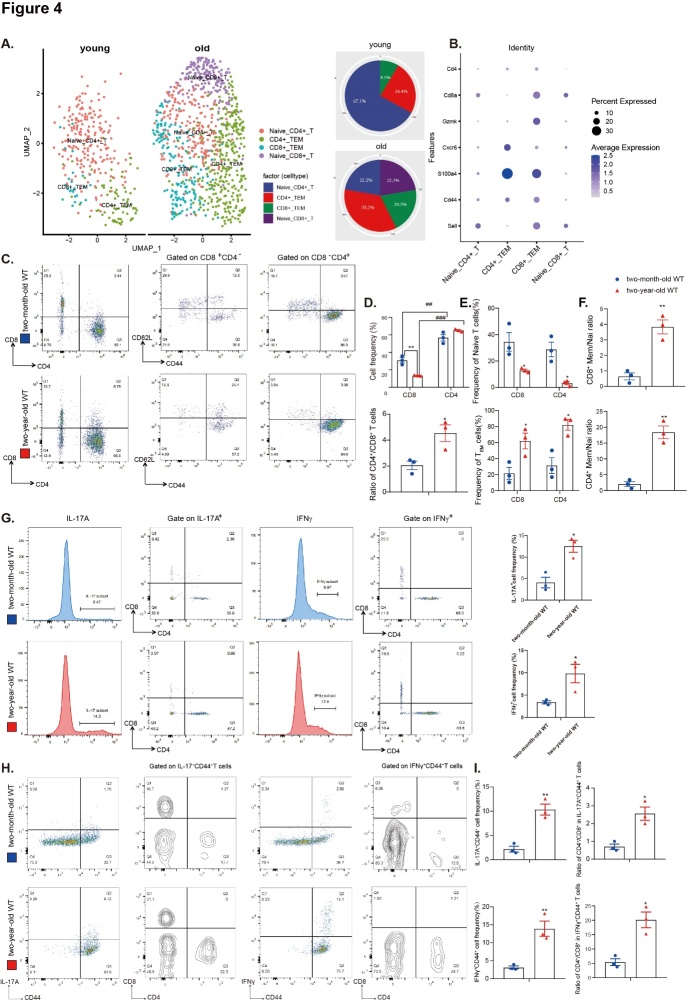
Physiological aging increases pulmonary CD4^+^ T_EM_ cells and IL-17A and IFNγ produced by CD4^+^ T cells. (A) UMAP plot showing the four main T cell subclusters identified in young and aged lungs. (B) Dot plot showing the signature gene expressions across the five subclusters. The size of the dots represents the proportion of cells expressing the specific marker, and the spectrum of color indicates the mean expression level of the markers. (C) Representative flow cytometric analyses of CD4 versus CD8 expression and of CD44 versus CD62L expression in the CD4^+^ T cell lineage or CD8^+^ T cell lineage in total lung cells from 2-month-old WT and 2-year-old WT mice. (D) Cell frequency of CD8^+^ T cells and CD4^+^ T cells in pulmonary live T cells and the ratio of CD4^+^ T cells versus CD8^+^ T cells. (E) Frequency of naïve T cells and T_EM_ cells in CD4^+^ T cells and CD8^+^ T cells. (F) The ratio of T_EM_ versus naïve T cells in the CD4^+^ and CD8^+^ compartments. (G) Representative flow cytometric analyses of IL-17A^+^ and IFNγ^+^ T cells and of CD4 versus CD8 expression on IL-17A^+^ T cell lineage or IFNγ^+^ T cell lineage on total lung cells. Absolute cell numbers of IL-17A^+^ T cells and IFNγ^+^ T cells are shown. (H) Representative flow cytometric analyses of CD4 and CD8 expression in the IL-17^+^CD44^+^ T cell lineage or IFNγ^+^CD44^+^ T cell lineage in total lung cells. (I) Representative flow cytometric analyses of IL-17A^+^CD44^+^ and IFNγ^+^CD44^+^ T cells and of CD4 versus CD8 expression in IL-17A^+^CD44^+^ T cell lineage or IFNγ^+^CD44^+^ T cell lineage in total lung cells. Three biological replicates were used per experiment (N = 3). Values are the mean ± SEM of three determinations. *p < 0.05, **p < 0.01 compared with the 2-month-old WT group; ^##^p < 0.01, ^###^p < 0.001 compared with CD8^+^ T cells in the same age group. Statistical analysis was performed with Student’s t-test and one-way ANOVA.

### Physiological aging leads to pulmonary dysfunction and SAPF

We evaluated expiratory- and inspiratory-related indexes to assess pulmonary function. Peak inspiratory flow, tidal volume, minute volume, accumulated volume, expiratory flow 50, and ratio of expiration time decreased significantly in 2-year-old physiologically aged WT mice compared with 2-month-old young WT mice. This contrasts the dramatically increased inspiration time, expiration time, relaxation time, and end-expiratory pause in the 2-year-old (physiologically aged) WT mice compared with the young (2-month-old) WT mice (Fig. 2A-K). These results indicate that physiological aging causes notable pulmonary dysfunction.

H&E staining revealed significantly increased infiltration of inflammatory cells, senescence-associated-β-galactosidase (SA-β-gal)-positive areas, p16- and p53-positive cells or proteins, CD3e-positive cells, and IFNγ positive cells/areas or proteins in the lungs of 2-year-old physiologically aged WT mice compared to 2-month-old (young) WT mice (Fig. 2L-M and Supplementary Fig. 2A-D).

To investigate whether physiological aging leads to pulmonary fibrosis, the lungs were examined for fibrosis markers using Masson’s trichrome staining (Masson), immunohistochemistry (IHC) staining, and Western blots. An increase was shown in Masson-labeled interstitial fibers, the percentage of α-smooth muscle actin (α-SMA)-, type Ι collagen (collagen 1)-, TGF-β1-, IL-11-, and IL-11Rα1-positive cells or areas, and the expression of α-SMA and collagen 1. However, an obvious decrease in the expression of SFTPC was shown in the lungs of 2-year-old aged WT mice compared with 2-month-old WT mice (Fig. 2L-M and Supplementary Fig. 2C-D).

### IL-17A and IFNγ signaling pathways are activated in physiologically aged lungs

All the genes expressed between young and old lungs were sorted according to the value of logFC to perform an unbiased GSEA analysis by the Cluster Profiler R package. Disease enrichment analysis showed significant enrichment in the IL-17 signaling pathway, IFNγ response [23], immune response activation, and collagen-containing extracellular matrix in aged lungs compared to young lungs (Supplementary Fig. 1C). The lungs were examined for IL-17A and IFNγ using Western blots and IHC stains to investigate whether physiological aging led to activation of the IL-17A and IFN-γ signaling pathways. IL-17A and IFNγ protein expression and pulmonary CD3e-positive, IL-17A-positive, and IFNγ-positive cells or areas were increased in the lungs of 2-year-old physiologically aged WT mice compared with 2-month-old WT mice (Supplementary Fig. 2C-F and Fig. 2L-M).

### Senescence, SASP, and activation of T cells occur in physiologically aged lungs

Gene set scoring was used to evaluate SASP-related gene set scores between young and old lungs based on the dataset GSE124872 [23]. The results showed that SASP-related genes were augmented in seven cell types in aged lungs. These cells included T cells, alveolar macrophages, ciliated cells, goblet cells, conventional dendritic cells, plasma cells, and natural killer cells (Fig. 3A-B), illustrating that the physiologically aged lung is characterized by cellular stress and chronic inflammation. Cellular senescence assessment showed that pulmonary aging is driven by aged T cells, alveolar macrophages, ciliated cells, conventional dendritic cells, natural killer cells, plasma cells, and plasmacytoid dendritic cells (Supplementary Fig. 3A). The average expression of aging-related genes *p19(cdkn2d)*, *p21(cdkn1a)*, and *p27(cdkn1b)* were upregulated in physiologically aged lung compared to young lung (Fig. 3C). SASP-related genes *Cxcl15(IL-8)*, *IL-1β*, and *Ifnγ* were upregulated in physiologically aged lung (Fig. 3D). DEGs of young and aged T cells were used for KEGG enrichment analysis and GO-BP enrichment analysis. The results showed that T cells and receptor signaling were activated in the lungs of 2-year-old physiologically aged WT mice compared to 2-month-old WT mice (Supplementary Fig. 3B-C).

**Figure 5.**
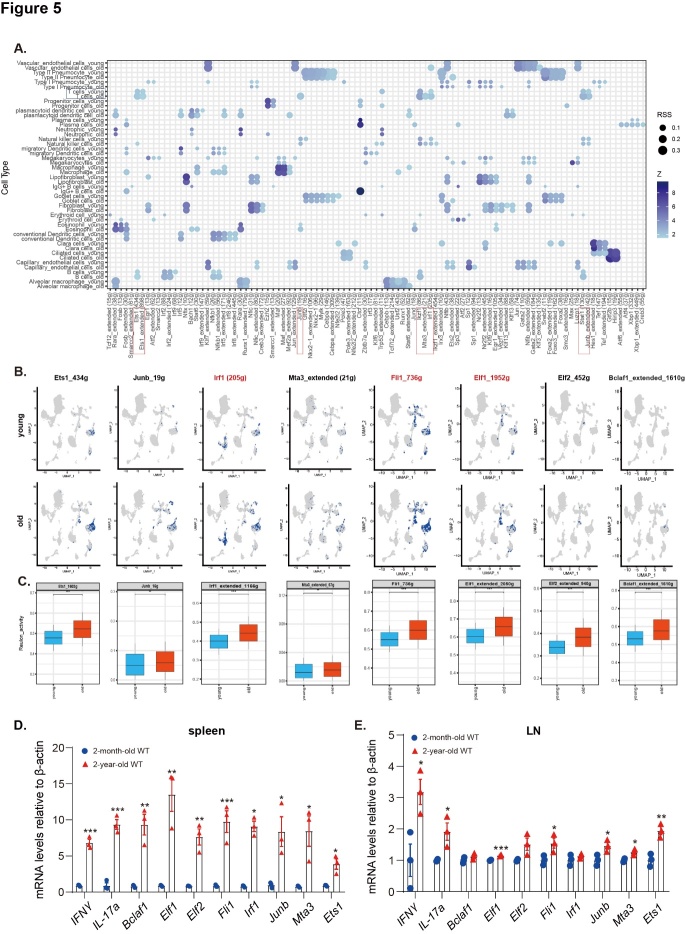
Specific regulon activity increases in T cell subclusters with aging. (A) The “signature genes” in the X-axis are the set of transcription factors and their downstream target genes. Dot plots show the expression levels of these “signature genes” across the 24 cellular clusters. The size of the dots represents the proportion of regulon in specific cell types, and the spectrum of color indicates the Z-score. (B) Feature plots showing specific regulon activity (*Ets1*, *Junb*, *Irf1*, *Mta3*, *Fli1*, *Elf1*, *Elf2* or *Bclaf1*) in T cell subclusters. (C) Expression values are represented as normalized log_2_-transformed counts. Values are the mean ± SD. *p < 0.05, **p < 0.01, ***p < 0.001 compared with the young group (two-sided Wilcoxon rank-sum tests). (D) *Ifnγ*, *Il-17a*, *Bclaf1*, *Elf1*, *Elf2*, *Fli1*, *Irf1*, *Junb*, *Mta3*, and *Ets1* mRNA levels in the spleen and mediastinal lymph nodes by real-time RT-PCR, calculated as the ratio to β-actin mRNA and expressed relative to the control. The mRNA relative expression was normalized to the 2-month-old group. Values are the mean ± SEM of three determinations. *p < 0.05, **p < 0.01, ***p < 0.001 compared with the 2-month-old group. Statistical analysis was performed with Student’s t-test.

### Physiological aging increases pulmonary CD4^+^ T_EM_ cells, and IL-17A and IFNγ are produced mainly by CD4^+^ T_EM_ cells

To understand the composition of the T cell subsets in aging pulmonary tissues, we further defined the subpopulations of T cells by a group of signature genes that were summarized from several studies [47, 48]. Based on the UMAP algorithm, four distinct subclusters of T cells were identified, including naïve CD4^+^ T, CD4^+^ T_EM_, CD8^+^ T_EM_, and naïve CD8^+^ T cells (Fig. 4A-B). This result showed that the number of CD4^+^ T_EM_, CD8^+^ T_EM_, and naïve CD8^+^ T cells increased with aging while naïve CD4^+^ T cells decreased (Fig. 4A-B).

Next, the subtypes and function of T cells were investigated in lung tissues from 2-year-old physiologically aged WT mice and 2-month-old WT mice. Aging decreased the frequency of total pulmonary live CD8^+^ cells but increased the frequency of total pulmonary live CD4^+^ cells and the ratio of total pulmonary live CD4^+^ T cells relative to total pulmonary live CD8^+^ T cells. CD4^+^ T cells accounted for most of the pulmonary lymphocytes and increased with aging (Fig. 4C-D). Aging also caused a decreased output of CD4^+^ naïve and CD8^+^ naïve T cells, an increased production of CD4^+^ T_EM_ and CD8^+^ T_EM_ cells, and an increased ratio of T_EM_ cells relative to naïve cells (Fig. 4E-F). Aging caused an increased frequency of IL-17A-positive and IFNγ-positive cells (Fig. 4G). CD4^+^ T_EM_ cells were the main cells producing IL-17A and IFNγ compared to CD8^+^ T_EM_ cells (Fig. 4G-I). An analysis of single-cell data also revealed that IFNγ was secreted mainly by T cells (Supplementary Fig. 5A). It has been shown that with age, senescent T cells accumulate in the spleen, which contributes to the decline in immune function [49]. Therefore, splenic cells were detected by western blots for aging-related molecules, i.e., IL-17A and IFNγ. An increase was shown in the expression of p19, p21, p53, IL-17A, and IFNγ in spleens of 2-year-old physiologically aged WT mice compared with 2-month-old WT mice (Supplementary Fig. 4A-B). The flow cytometry results showed that the proportion of CD4^+^ T_EM_ and CD8^+^ T_EM_ in spleens was increased (Supplementary Fig. 4, C-D), which was consistent with a previous study [50].

### Physiological aging increases pulmonary cell responsiveness to IFNγ signaling

The effect of interferons in the defense against pathogens has been explained [51]. However, the effect of interferon on pulmonary cells has not been reported. When pathogens invade the body through the lungs, interferon is released by the lung tissue to quickly clear the threat. However, the effect of IFNγ on lung cells has remained unclear. Analyses of the single-cell data revealed that some cell types in lung tissues showed an age-associated increase in the expression of the IFNγ receptors *Ifnγr1* and *Ifnγr2* (Supplementary Fig. 6B and Supplementary Fig. 5B-C) and a strong response to IFNγ signaling (Supplementary Fig. 6A), especially for T cells and AT2 cells. *Ifnγr1* was more widely expressed than *Ifnγr2*. Next, the responsiveness to IFNγ signaling between young and old pulmonary cells was compared using GSEA analysis and gene set score analysis. The results showed that some pulmonary cells, especially AT2 cells and T cells, responded to IFNγ signaling, and the responsiveness significantly increased during aging (Supplementary Fig. 6C). They also expressed the IFNα receptors *Ifnαr1* and *Ifnαr2* and responded to IFNα signaling (Supplementary Fig. 5D-F).

### Specific regulon activity increases in T cell subclusters, and IFNγ and IL-17a are transcriptionally upregulated in pulmonary T cells with aging

Using the SCENIC [28] R package, transcription factors that might transcriptionally regulate IFNγ in pulmonary cells and T cells between young and physiologically aged mice were analyzed based on the dataset GSE124872 [23]. The results showed that IFNγ was the target of several transcription factors whose transcriptional activities might be upregulated in pulmonary T cells of physiologically aged mice compared to young mice. These transcription factors included *Ets1*, *Junb*, *Irf1*, *Ikzf1*, *Mta3*, and *Stat1* (Fig. 5A).

Transcription factors and their target genes together make up the regulon [28]. Next, whether these regulon activities were upregulated with aging was investigated. The results showed that the regulon activity of *Ets1*, *Junb*, *Irf1*, *Mta3*, *Fli1*, *Elf1*, *Elf2*, and *Bclaf1* was upregulated in pulmonary T cells of physiologically aged mice compared to young mice (Fig. 5B-C). However, there was no difference between the regulon activity of *Stat1* or *Ikzf1* (Supplementary Fig. 7A-B). The mRNA levels of these transcription factors and *IFNγ* and *IL-17a* in peripheral immune organs, including the spleen and mediastinal lymph nodes, were investigated. The expression of *IFN-γ*, *IL-17a*, *Bclaf1*, *Elf1*, *Elf2*, *Fli1*, *Irf1*, *Junb*, *Mta3*, and *Ets1* was increased in the spleens, and the expression of *IFN-γ*, *IL-17a*, *Elf1*, *Fli1*, *Junb*, *Mta3*, and *Ets1* was increased in the mediastinal lymph nodes of 2-year-old physiologically aged WT mice compared with 2-month-old WT mice (Fig. 5D-E).

**Figure 6.**
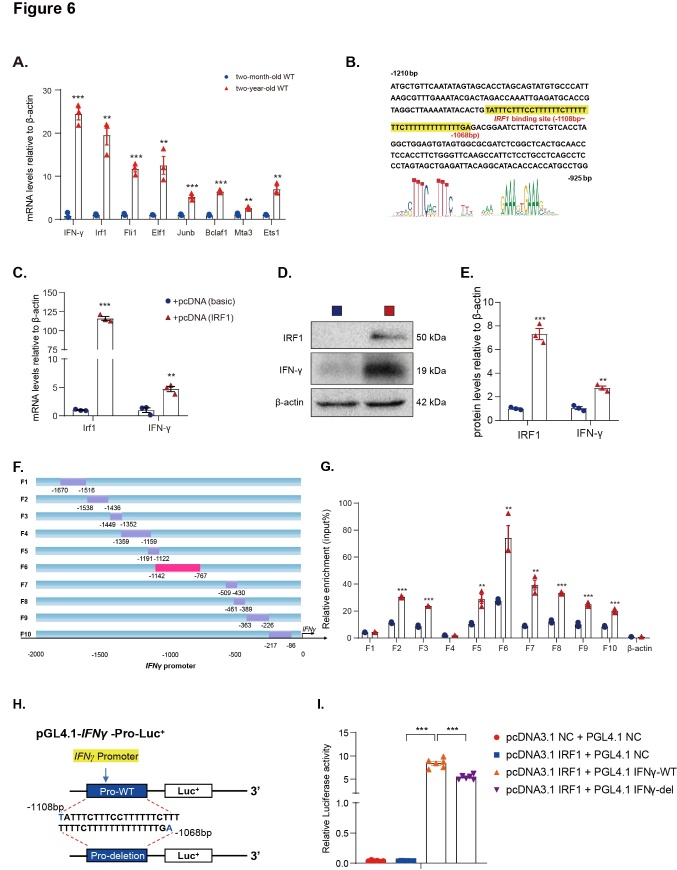
*IFNγ* transcribed by IRF1 increases in pulmonary T cells with aging. (A) *Ifnγ*, *Irf1*, *Fli1, Elf1*, *Junb*, *Bclaf1*, *Mta3*, and *Ets1* mRNA levels in CD4^+^ T_EM_ sorted from 2-month-old WT and 2-year-old WT mouse spleens by real-time RT-PCR, calculated as a ratio to *β-actin* mRNA and expressed relative to control. The mRNA relative expression was normalized to the basic-transfected group. Three biological replicates were used per experiment. Values are the mean ± SEM of three determinations. *p < 0.05, **p < 0.01, ***p < 0.001 compared with the 2-month-old group. Statistical analysis was performed with one-way ANOVA. (B) IRF1-like sequence binding site (highlighted in yellow) in the *IFNγ* promoter region and the IRF1 sequence highlighted in red. (C) *IRF1* and *IFNγ* mRNA levels in 293T cells transfected with basic and IRF1-overexpression plasmid by real-time RT-PCR, calculated as a ratio to β-actin mRNA and expressed relative to control. The mRNA relative expression was normalized to the basic-transfected group. (D) Western blots of 293T cell extracts transfected with basic and *IRF1*-overexpression plasmid showing IRF1 and IFNγ. β-actin was the loading control. (E) Protein levels relative to β-actin were assessed by densitometric analysis and normalized to the basic-transfected group. Three biological replicates were used per experiment (N = 3). Values are the mean ± SEM of six determinations. (F) A model of IFN-γ promoter truncated primers. (G) ChIP assays were performed with chromatin prepared from 293T cells transfected with basic and IRF1-overexpression plasmid. The chromatin was immunoprecipitated with normal rabbit IgG or antibodies against IRF1, and precipitated genomic DNA was analyzed as relative enrichment through real-time PCR using different primers for the different regions of the *IFNγ* promoter. The β-actin promoter (-393 to -175 bp) was used as a negative control. Three biological replicates were used per experiment (N = 3). Values are the mean ± SEM of six determinations. **p < 0.01, ***p < 0.001 compared with the basic-transfected group. Statistical analysis was performed with Student’s t-test. (H) Schematic diagram of the structure of the *pGL4.10-IFNγ* promoter reporter plasmid and deletion construct of the *pGL4.10-IFNγ* promoter reporter plasmid. (I) *IFNγ* promoter activity was measured by a luciferase reporter gene assay. Six biological replicates were used per experiment (N = 6). Values are the mean ± SEM of three determinations. ***p < 0.001 compared with the corresponding group. Statistical analysis was performed with one-way ANOVA.

### IFNγ transcribed by IRF1 increases in pulmonary T cells with aging

CD4^+^ T_EM_ was sorted from the spleens of 2-month-old and 2-year-old physiologically aged WT mice by flow cytometry and the mRNA levels of the differentially expressed transcription factors were detected. *IRF1* had the highest expression among all the transcription factors in the 2-year-old physiologically aged WT mice (Fig. 6A). To testify whether IFNγ expression is regulated by IRF1, an *IRF1*-like sequence was identified in 2000 bp upstream of the *IFNγ* gene (JASPAR CORE database; http://jaspar.genereg.net/) (Fig. 6B). Following transfection of HEK293T cells with or without *IRF1* overexpression plasmid, the mRNA level of *IFNγ* in HEK293T cells increased (Fig. 6C). The protein level of IFNγ was also upregulated, suggesting that IRF1 regulated the expression of *IFNγ* (Fig. 6D-E).

To identify whether IRF1 binds to the promoter of *IFNγ*, a series of mouse *IRF1* promoter primers were constructed (Fig. 6F). Chromatin immunoprecipitation (ChIP) of HEK293T cells transfected with or without IRF1 was conducted. Anti-IRF1 primary antibody was used to capture the chromatin. The ChIP results showed that IRF1 significantly upregulated *IFNγ* in the region from -1142 to -767 bp compared with the control group (Fig. 6G). Next, to further clarify whether *IFNγ* expression is regulated by IRF1 at the transcriptional level, *IRF1*-overexpressed plasmid was transfected into HEK293T cells. Human *IFNγ* promoter luciferase reporter plasmids with or without an IRF1-like sequence (-1108~-1068 bp) deletion were constructed (Fig. 6H). The results of the luciferase activity assay demonstrated that luciferase expression levels were increased significantly in HEK293T cells transfected with an IRF1 binding sequence plasmid compared with the vehicle group. In contrast, luciferase activity decreased obviously in HEK293T cells transfected with a pGL4.1-*IFNγ*-deletion plasmid compared with an IRF1 binding sequence plasmid (Fig. 6I). Therefore, IRF1 binds to the promoter of *IFNγ* and regulates *IFNγ* expression at the transcriptional level.

### IFNγ produced by pulmonary CD4^+^ T_EM_ cells promotes senescence and epithelial-to-mesenchymal transition of AT2 cells with aging

**Figure 7.**
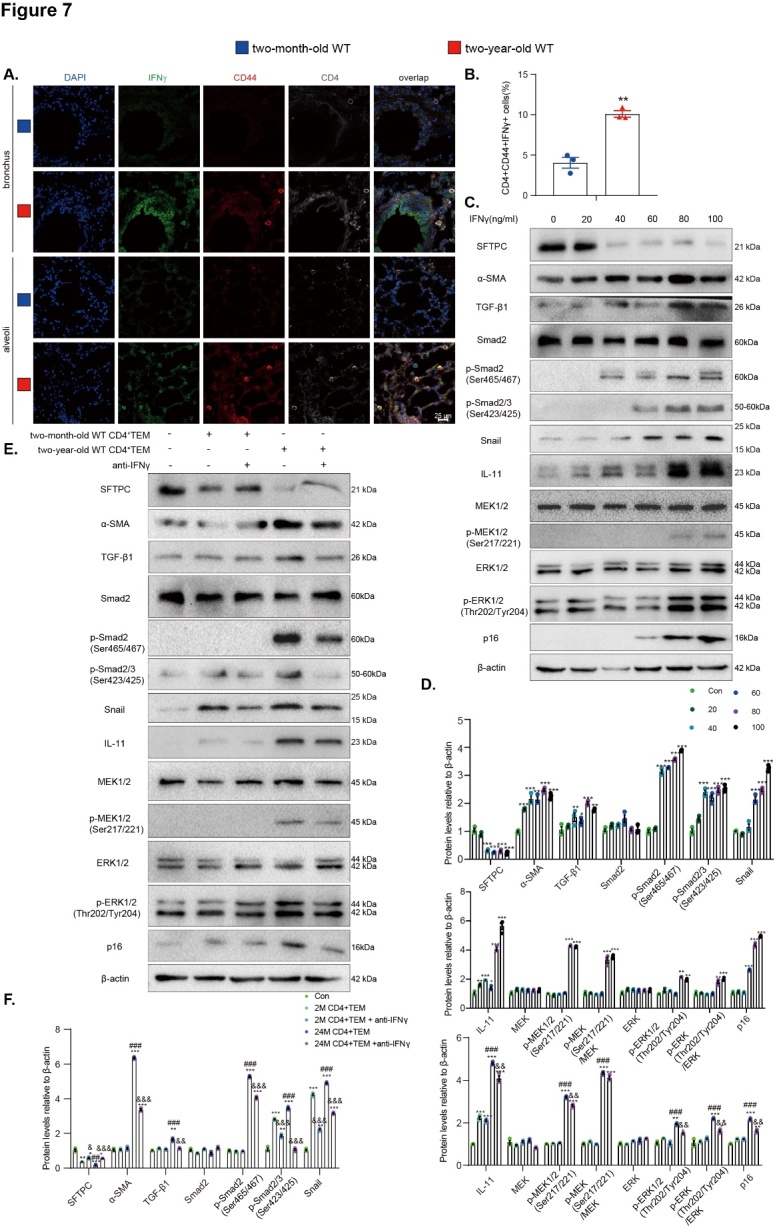
IFNγ produced by pulmonary CD4^+^ T_EM_ cells promotes senescence and epithelial-to-mesenchymal transition of AT2 cells with aging. (A) Representative micrographs of pulmonary cells immunofluorescently stained for CD4, CD44, and IFNγ with DAPI staining the nucleus. (B) The percentage of CD4-, CD44-, and IFNγ-positive cells. **p < 0.01 compared with the 2-month-old group. Statistical analysis was performed with Student’s t-test. (C) Western blots showing SFTPC, α-SMA, TGF-β1, Smad2, p-Smad2(Ser465/467), p-Smad2/3(Ser423/425), Snail, IL-11, MEK1/2, p-MEK1/2 (Ser217/221), ERK1/2, p-ERK1/2(Thr202/Tyr204), and p16 in AT2 cells treated with different concentrations (0, 20, 40, 60, 80, and 100 ng/mL) of IFNγ for 48 h. β-actin was used as the loading control. (D) Protein levels relative to β-actin were assessed by densitometric analysis and normalized to the 0 ng/mL IFNγ-treated group. Three biological replicates were used per experiment (N = 3). Values are the mean ± SEM of six determinations. *p < 0.05, **p < 0.01, ***p < 0.001 compared with 0 ng/mL IFNγ-treated group. (E) Western blots showing SFTPC, α-SMA, TGF-β1, Smad2, p-Smad2(Ser465/467), p-Smad2/3(Ser423/425), Snail, IL-11, MEK1/2, p-MEK1/2(Ser217/221), ERK1/2, p-ERK1/2(Thr202/Tyr204), and p16 in AT2 cells treated with different groups of sorted cells, IFNγ, and anti-IFNγ. β-actin was used as the loading control. (F) Protein levels relative to β-actin were assessed by densitometric analysis and normalized to the untreated group. Three biological replicates were used per experiment (N = 3). Values are the mean ± SEM of three determinations. *p < 0.05, **p < 0.01, ***p < 0.001 compared with the untreated group; ^#^p < 0.05, ^##^p < 0.01, ^###^p < 0.001 compared with the 2-month-old CD4^+^ T_EM_ cell treated group; ^&^p < 0.05, ^&&^p < 0.01, ^&&&^p < 0.001 compared with the CD4^+^ T_EM_ cell treated group. Statistical analysis was performed with one-way ANOVA.

By using immunofluorescence, we further confirmed that the ratio of pulmonary CD4^+^ T_EM_ cells increased, and the IFNγ produced by these cells was upregulated in 2-year-old physiologically aged WT mice compared with 2-month-old WT mice (Fig. 7A-B). AT2 cells were then treated with gradient concentrations of IFNγ (0, 20, 40, 60, 80, and 100 ng/mL). The results showed that the expression of α-SMA, p16, and TIME signaling proteins TGF-β1, Smad2, p-Smad2(Ser465/467), p-Smad2/3 (Ser423/425), Snail, IL-11, MEK1/2, p-MEK1/2 (Ser217/221), ERK1/2, and p-ERK1/2(Thr202/Tyr204) in AT2 cells was upregulated with the elevation of IFNγ concentration. In contrast, a significant decrease was observed in the expression of SFTPC (Fig. 7C-D). These results demonstrated that IFNγ promoted senescence and EMT of the AT2 cells.

Previous results have confirmed that the spleen of aged mice accumulate T_EM_ and produce a large amount of IFNγ (Supplementary Fig. 4A-D). According to a previous study, splenocyte transplantation from aged mice induces the senescence of multiple organs [52]. Therefore, in this study, CD4^+^ T_EM_ were sorted from the spleens of 2-month-old WT mice and 2-year-old physiologically aged WT mice by flow cytometry. These sorted cells were co-cultured with AT2 cells for 48 h to clarify whether the increased IFNγ produced by CD4^+^ T_EM_-induced senescence and EMT of AT2 cells with aging. The anti-IFNγ antibody was a kind of rescue. The expression of the above proteins related to senescence and TIME signaling were upregulated in AT2 cells co-cultured with CD4^+^ T_EM_ from 2-year-old mice. However, the level of SFTPC decreased noticeably, an effect that could be reversed by the addition of anti-IFNγ antibody (Fig. 7E-F). These results indicated that IFNγ produced by CD4^+^ T_EM_ promoted senescence and EMT of AT2 cells.

### Accumulated IRF1^+^CD4^+^ T_EM_ produces IFNγ in aging lungs, and anti-IRF1 primary antibody treatment inhibits the expression of IFNγ

Immunofluorescence staining for CD4, CD44, IRF1, and IFNγ was conducted to investigate the role of IRF1 in pulmonary CD4^+^ T_EM_ cells. The results showed that IRF1^+^CD4^+^ T_EM_ cells accumulated in the lung with aging, and subsequently, the IFNγ produced by these cells increased in 2-year-old WT mice compared with 2-month-old WT mice (Supplementary Fig. 8A-D). CD4^+^ T_EM_ cells were then sorted from the lungs of 2-month-old WT mice and 2-year-old physiologically aged WT mice and treated with anti-IRF1 antibody for 48 h. The expression of IFNγ in pulmonary CD4^+^ T_EM_ cells decreased following the administration of anti-IRF1 antibody (Supplementary Fig. 8E-F). These results illustrated that IRF1 positively regulated IFNγ in pulmonary CD4^+^ T_EM_ cells, which then affected pulmonary function as mentioned above.

### Differentiation dynamics of T cells with aging

**Figure 8.**
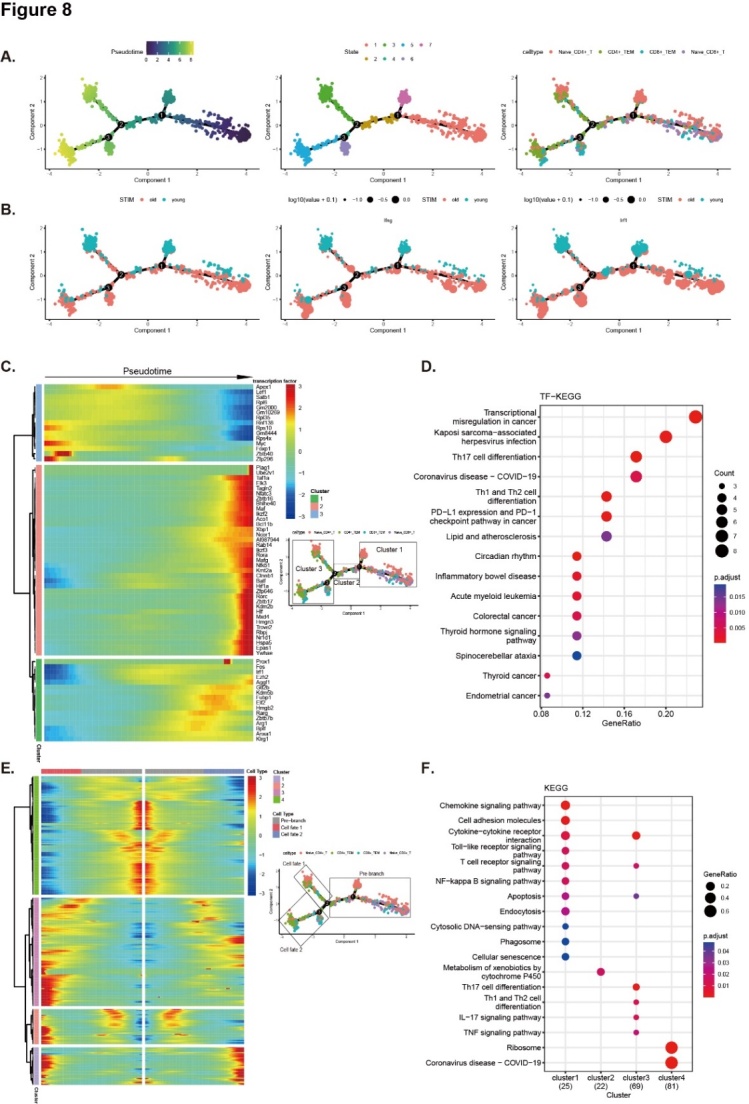
Differentiation dynamics of T cells with aging. Pseudotemporal trajectories identified transcriptional dynamics of T cells. (A) Monocle pseudotime analysis revealing the progression of the seven stages of T cell differentiation, which included the four T cell subtypes (naïve CD4^+^, CD4^+^ T_EM_, naïve CD8^+^, and CD8^+^ T_EM_). (B) Expression levels of IRF1 and IFNγ in differentiation trajectories, the red point indicating the cells of the older mice and the blue point indicating the cells of the young mice. Dot size indicates the expression levels. Values are log10 (value + 0.1). (C) Heatmap showing the scaled expression of differentially expressed transcription factor genes along with the pseudotime, cataloged into three major gene clusters. According to pseudotemporal trajectories illustrated by (A) and (B), clusters 1 to 3 were used to label the specific branch. The box was used to choose a branch point to inspect in illustrations. (D) Pathway enrichment analysis associated with transcription factor genes as in (C). (E) Heatmap showing the scaled expression of differently expressed genes in the four branches. From the center to the left of the heatmap, the kinetic curve from the root along the trajectory to CD4^+^ T_EM_. From the center to the right, the curve from the root to CD8^+^ T_EM_. According to pseudotemporal trajectories illustrated by (A) and (B), pre-branch, cell fate 1, and cell fate 2 were used to label the specific branch. The genes were clustered hierarchically, and clusters 1 to 4 cells were automatically classified according to similar lineage-dependent gene expression patterns. The box was used to choose a branch point to inspect in illustrations. (F) KEGG analysis of differently expressed genes associated with three gene clusters as in (E).

To reveal the differentiation dynamics of the T cells, the developmental trajectory of T cells was reconstructed using the dataset GSE124872 [23]. Ordering the cells in pseudotime arranged T cells into a major trajectory with three bifurcations and seven stages. Four clusters of T cells were found at different stages of differentiation. Naïve T cells were located toward the origin of the trajectory, while memory T cells were found near the terminal end of the trajectory, which partly served as a validation for the constructed trajectory (Fig. 8A). We also investigated the expression levels of IRF1 and IFNγ, and the results showed that IRF1 and IFNγ were expressed mainly in senescent T cells (Fig. 8B).

To determine which transcription factors were driving T cell differentiation, pseudotime dynamics of significantly changed transcription factors among the four subclusters were examined. They were arranged into three modules according to their pseudotemporal expression patterns. Interestingly, the gene set enrichment analysis for these transcription factors revealed that various signaling pathways, including Th17-, Th1-, and Th2-cell differentiation, were involved in the differentiation process of the T cells (Fig. 8C-D).

Since CD4^+^ T_EM_ and CD8^+^ T_EM_ cells were at two distinct branch terminal points at the end of the differentiation trajectory, the gene expression patterns involved in the continuum transition were further dissected. The cluster 1 and cluster 3 genes were involved in multiple KEGG terms (“T cell receptor signaling pathway”, “Toll-like receptor signaling pathway”, “NF-kappa B signaling pathway”, “Th17 cell differentiation”, “Th1 and Th2 cell differentiation”, “IL17 signaling pathway”, and “TNF signaling pathway”), implying that Th17, Th1, and Th2 cell differentiation and inflammatory signaling pathways are involved in the developmental trajectories of T cells (Fig. 8E-F).

### Pulmonary T cell interactions with other surrounding cells increase with aging

The CellChat package was applied to observe the communications of pulmonary cells and analyze communication differences among pulmonary cell populations between the young and physiologically aged mice. This package combines social network analysis, pattern recognition, and multiple learning methods to quantitatively describe and compare the inferred intercellular communication networks, as previously described [27].

The number of interactions and the interaction strength among T cells and endothelial cells (including capillary endothelial cells and vascular endothelial cells), macrophages (including alveolar macrophages and interstitial macrophages), epithelial cells (including AT1 and AT2 cells), erythroid cell, megakaryocytes, natural killer cells, granulocytes (including neutrophils and eosinophils), fibroblasts, progenitor cells, dendritic cells (including migratory dendritic cells, plasmacytoid dendritic cells, and conventional dendritic cells), and B cells (including plasma cells, Igha^+^ B cells, and conventional B cells) were significantly enhanced in the lungs of older mice compared to younger mice (Supplementary Fig. 8A). The incoming interaction strength was enhanced, and there was a slight increase in the T cells’ outgoing interaction strength, indicating that the T cells expressed more cell surface receptors and received stimulation by more ligands (Supplementary Fig. 8B).

The effect of pulmonary T cells on other cells through the secretion of IFNγ was investigated. The results showed that the pulmonary T cells affected other pulmonary cells via IFNγ signaling and was obviously enhanced in physiologically aged mice compared to young mice. Type II pneumocytes, alveolar macrophages, type I pneumocytes, migratory dendritic cells, plasmacytoid dendritic cells, macrophages, and conventional dendritic cells were affected (Supplementary Fig. 8C-D).

The differences in receptors and ligand signaling pathways among pulmonary cell populations between the young and the old were further analyzed. Fasl-fas signaling, which mediates T cell killing, increased from T cells to other cells. Multiple ligand-receptor signaling of T cells acting on epithelial cells was enhanced (including Tnfsf14-Ltbr, Tnf-Tnfrsf1a, Itgb2-Icam1, Lta-Tnfrsf1b, Mif- [Cd74 or Cd44]) (Supplementary Fig. 8D).

## DISCUSSION

This study found that SAPD was monitored by markers of alveolar epithelial cells and mediated by T cells. Furthermore, IFN-γ and IL-17A signaling pathways were activated, and senescence, SASP, and activation of T cells were shown in physiologically aged lungs. Physiological aging led to pulmonary dysfunction, cell senescence, and TIME signaling-mediated SAPF. Physiological aging increased pulmonary CD4^+^ T_EM_ cells. It was found that IL-17A and IFNγ were produced mainly by CD4^+^ T_EM_ cells, and pulmonary cells had increased responsiveness to IFNγ signaling. Specific regulon activity was increased in T cell subclusters. Increased secretions of IFNγ, transcribed mainly by IRF1 in CD4^+^ T_EM_ cells, promoted EMT of AT2 cells by activating TIME signaling. Aging might drive T cell differentiation toward helper T cells. Cell interactions of pulmonary T cells with other surrounding cells increased with aging. These factors worked together to aggravate SAPF and accelerate the process of SAPD.

Recent evidence has indicated that intrinsic alterations of CD4^+^ T cells contribute to chronic inflammation and induce an organism-wide aging phenotype. This supports the idea that T cell aging plays a major role in body-wide deterioration and lung function decline [6]. This study combined single-cell transcriptomics and a battery of verification experiments to study the role of T cells in physiologically aged lungs. It was found that the occurrence and development of chronic lung diseases were related to alveolar epithelial cell markers, such as *sftpa1*, *sftpb*, *sftpc*, and *sftpd*, implying that epithelial cell dysfunction might be associated with a deterioration in lung function. This study re-analyzed single-cell data and found that multiple cell types, including T cells, raised cellular senescence and SASP phenotype in aged lungs. Several lines of evidence have demonstrated that the accumulation of SASP factors could also account for pulmonary fibrosis and other chronic respiratory diseases [3, 22]. In this study, an increased number of total T cells and augmented expressions of aging- and SASP-related molecules were noted in T cells in aged lungs. T cell aging may be one of the principal manifestations of immunosenescence, which is characterized by increased memory T cells and the time-dependent loss of immune-system vigor with increased unwarranted overreactions that lead to autoinflammatory and autoimmune disease [6, 53, 54]. To further clarify the subset composition of senescent T cells, unsupervised clustering of T cells in aged lungs was performed. The results showed that T_EM_ cells (including CD4^+^ and CD8^+^) increased, which was consistent with previous reports of an increase in memory T cells and an upregulation of pro-inflammatory molecules during aging [55]. Indeed, with aging, the immune system of the elderly is remodeled with fewer naïve cells, an increase in dysfunctional memory cells, primary lymphoid organ involution, and an altered innate immune response, leading to greater susceptibility to infectious diseases and reduced response to vaccination [56]. The T_EM_ cells become part of the destructor to attack normal tissue and induce chronic inflammatory diseases [42, 57].

Further, this study investigated how the increased T_EM_ cells act as the destructor to attack normal tissue and induce chronic inflammatory diseases in aged lungs. A previous study demonstrated that IFNγ was upregulated in physiologically aged lungs [23], implying that IFNγ signaling might be linked to aging-related lung diseases. This study found that multiple cell types expressed the IFNγ receptor, including Ifnγr1 and Ifnγr2, and exhibited an age-associated increase in the IFNγ response signature in the lungs. IFNγ has been reported to adversely affect cell proliferation [18]. The previous study demonstrated that CD8^+^ T_EM_ cells develop under the impact of an aged environment, contributing to an inflammation phenotype via increased secretion of GZMK [58]. However, in our study, upregulated IFNγ was attributed mainly to the increased CD4^+^ T_EM_ in aged lungs. IFNγ is traditionally known as a cytokine against viral infections and has anti-tumorigenic activity. However, recent evidence indicated that IFNγ produced by T cells in old brains delayed the proliferation of neural stem cells through Ifngr1 and Ifngr2 mediation [18]. Aging-induced IL-17 and IFNγ can facilitate alveolar bone loss and osteoclast differentiation [59], while anti-IFNγ therapy can rescue acute lung injury [17]. These findings show that the negative effects of IFNγ on stromal cells should not be ignored. Which cells in the aging lung are sensitive to the IFNγ response and the role that IFNγ plays in the occurrence and process of SAPD remain unclear.

This study found that some cell types in the lung, especially the T cells and AT2 cells, showed an age-associated increase in the IFNγ receptors Ifnγr1 and Ifnγr2 and a strong response to IFNγ signaling. The dramatic decline in the number of AT2 cells in aged lungs may be attributed to increased IFNγ secreted by CD4^+^ T_EM_. Therefore, experiments were conducted showing that T cells or IFNγ facilitated EMT of AT2 cells and aging in a dose-dependent manner. These effects were mediated by TIME signaling, which was reversed by anti-IFNγ treatment. Moreover, cell communication analysis on single-cell datasets also verified the effects of T cells on epithelial cells through IFNγ signaling. These results suggested that the destructive effect of excessive IFNγ on the functional cells of tissues cannot be underestimated. Degradation of pulmonary function during aging was associated with massive secretion of IFNγ by CD4^+^ T_EM_ cells, although other interferons may also affect them.

To further clarify the regulatory mechanism of IFNγ production, a set of transcription factors (including *Ets1, Junb, Ikzf1, Mta3, Irf1*, and *Stat1*) exhibiting high activities was obtained. The high activities imply that they might play an important role in T cell biological function. *IFNγ* was found to be a target of Irf1. According to a previous study, Irf1 mediates IFN-I (IFN-α/β) and IFN-III (IFN-λ) production [60]. However, the regulation of Irf1 on IFNγ has not been discovered. The ChIP results of the current study with the dual luciferase assay showed that Irf1 could promote the expression of *IFNγ* by binding to the promoter of *IFNγ*.

A previous study found that *IRF-1* gene global knockout mice produced lower levels of IFNγ than WT mice [61]. In this article, IFNγ transcriptionally regulated by IRF1 in CD4^+^ T_EM_ cells promoted epithelial-to-mesenchymal transition by activating TIME signaling and cell senescence of AT2 cells with aging. This caused the pathogenesis of SAPF and pulmonary dysfunction in physiologically aged mice. Anti-IRF1 antibody was applied to treat pulmonary CD4^+^ T_EM_, which inhibited the expression of IFNγ. This suggests that anti-IRF1 antibody could ameliorate the pathogenesis of SAPF and pulmonary dysfunction in physiologically aged mice by inhibiting the expression of IFNγ. Whether *IRF1*-conditioned knockout or specific overexpression regulates the pathogenesis of SAPF needs further study.

The senescent T cells directly reach the final differentiation stage, resulting in decreased ability to respond to antigens and reduced differentiation plasticity [6]. This study analyzed the developmental trajectory of T cells and selected transcription factors involved in trajectory development for KEGG analysis. These transcription factors were found to be involved mainly in the differentiation process of Th1, Th2, and Th17 cells. Th1 cells are widely believed to be the main source of IFNγ [62]. Furthermore, CD4^+^ T_EM_ cells and CD8^+^ T_EM_ cells were found to follow different developmental trajectories, and the differences between CD4^+^ T_EM_ cells and CD8^+^ T_EM_ differentiation involved multiple inflammatory signaling pathways. These findings suggested that naïve T cells differentiated mainly into Th cells and thus adversely affected the stromal cells by producing IFNγ in the lung during aging.

In summary, this study demonstrated that IFNγ transcribed by IRF1 in CD4^+^ T_EM_ cells promoted EMT of AT2 cells by activating TIME signaling in physiologically aged lungs. Therefore, CD4^+^ T_EM_ and IFNγ transcribed by IRF1 could be therapeutic targets for preventing SAPF.

## Supplementary Materials

The Supplementary data can be found online at: www.aginganddisease.org/EN/10.14336/AD.2023.0320.
