# Supplementary material for: IFNγ Transcribed by IRF1 in CD4+ Effector Memory T Cells Promotes Senescence-Associated Pulmonary Fibrosis
Source: Aging Dis. 2023 Dec 1;14(6):2215–37. doi: 10.14336/AD.2023.0320 (PMC10676796; doi:10.14336/AD.2023.0320)
Supplement: Supplementary file 1 — The Supplementary data can be found online at: www.aginganddisease.org/EN/10.14336/AD.2023.0320. [file AD-14-6-2215-s.pdf]

SUPPLEMENTARY DATA

IFNγ Transcribed by IRF1 in $CD4^+$ Effector Memory T Cells Promotes Senescence-Associated Pulmonary Fibrosis

Haiyun Chen[1,2,4#], Qiuyi Wang[1#], Jie Li[3#], Yuan Li [5#], Ao Chen[1], Jiawen Zhou[1], Jingyu Zhao[1], Zhiyuan Mao[1], Zihao Zhou[1], Jin'ge Zhang[1], Yue Wang[1], Rong Wang[1], Qing Li[6], Yongjie Zhang[1], Runqiu Jiang[2], Dengshun Miao[1,4], Jianliang Jin[1*]

© 2023. Chen H et al. Published online at http://www.aginganddisease.org/EN/10.14336/AD.2023.0320

SUPPLEMENTARY DATA

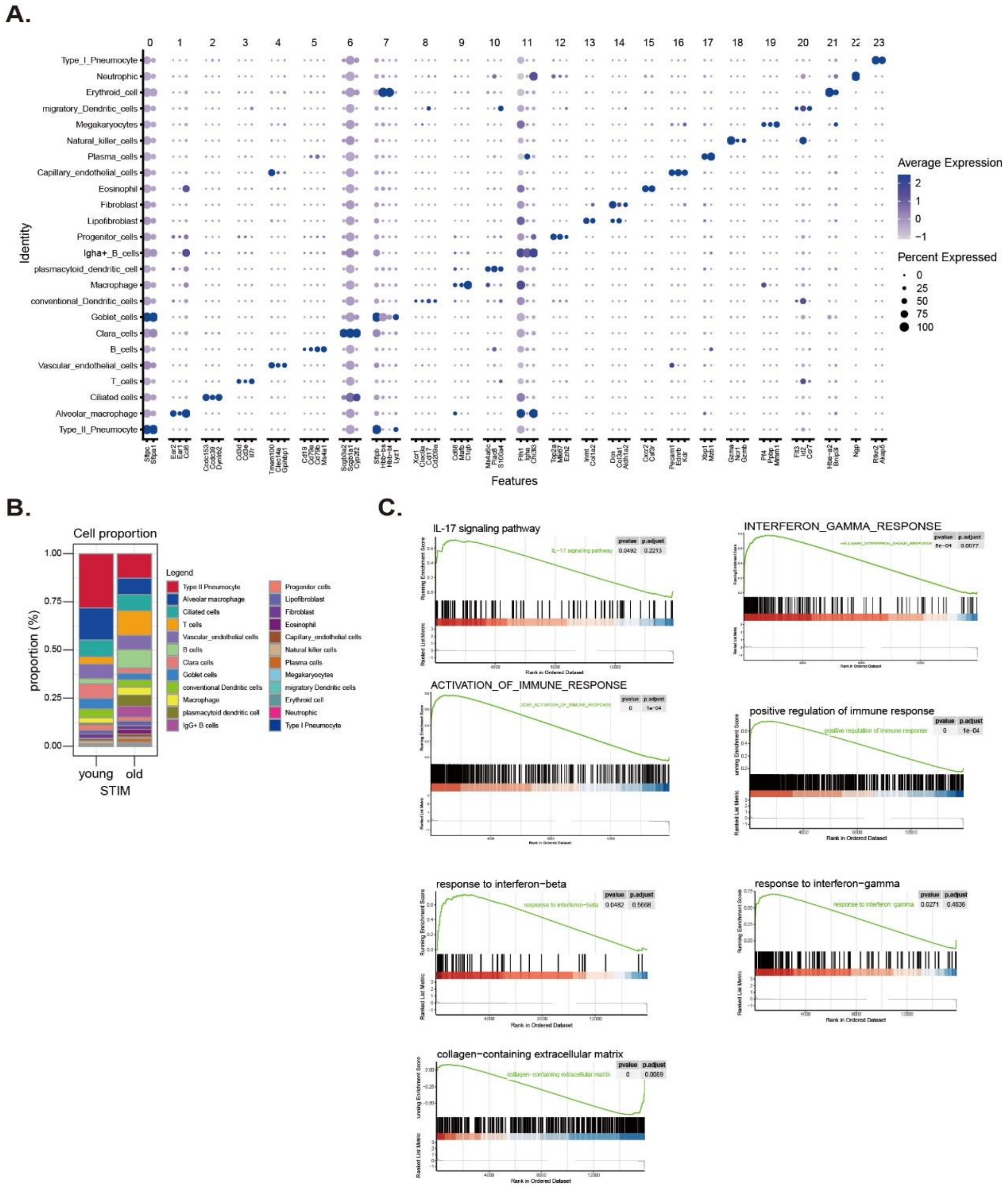

Supplementary Figure 1. Activation of IL-17A and IFN-γ signaling pathways shown in physiologically aged pulmonary cells. (A) Dot plots showing the signature gene expressions across the 24 cellular clusters. The size of the dots represents the proportion of cells expressing the specific markers, and the spectrum of color indicates the mean expression levels of the markers. (B) The proportion of different cell types in the lung tissue of young and aged mice. (C) Genes negatively associated with IL-17 signaling pathway, interferon-gamma (IFN-γ) response, immune response activation and collagen-containing extracellular matrix, are downregulated in aged lung tissues, as identified by gene set enrichment analysis (GSEA).

© 2023. Chen H et al. Published online at http://www.aginganddisease.org/EN/10.14336/AD.2023.0320

SUPPLEMENTARY DATA

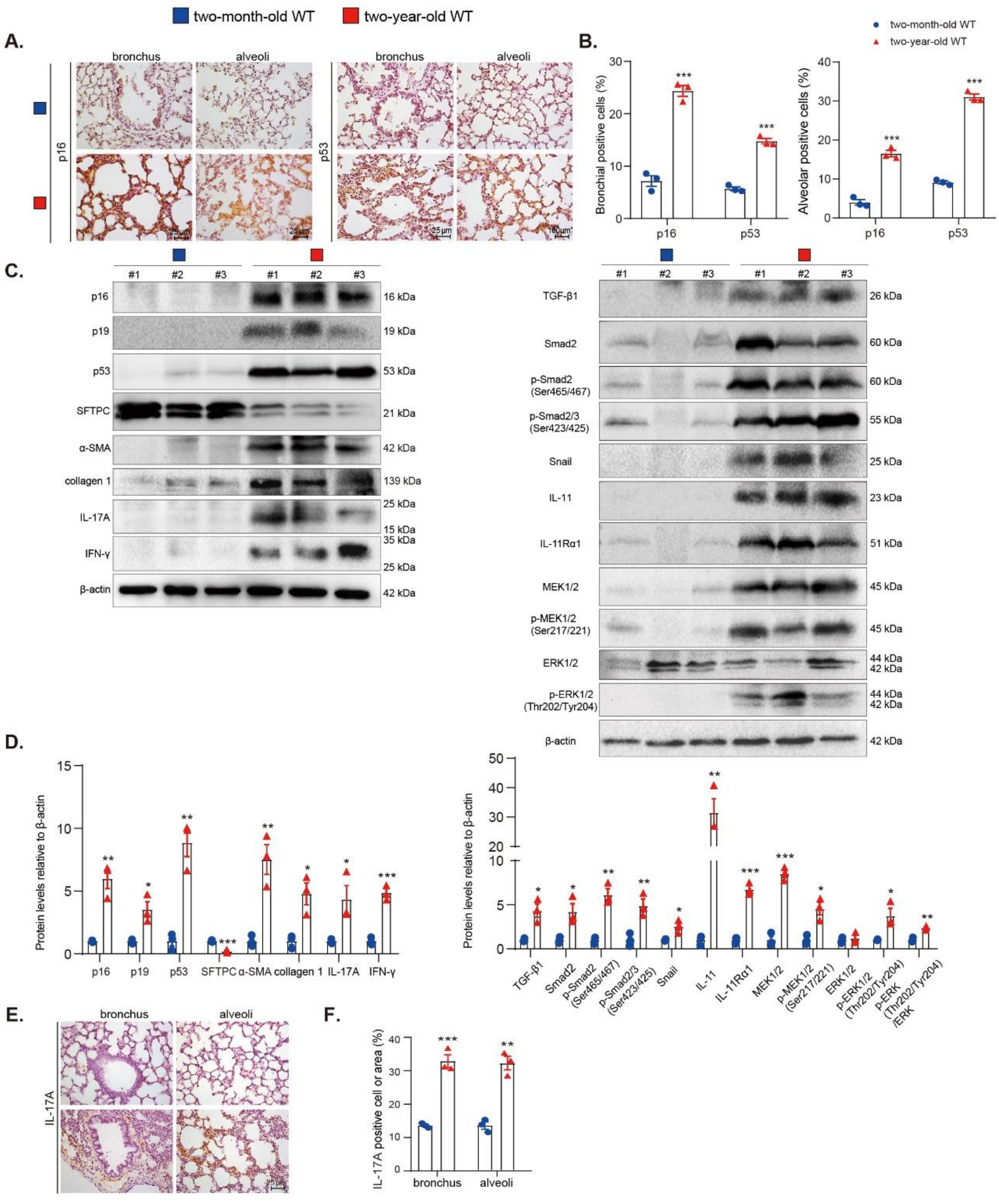

Supplementary Figure 2. Physiological aging leads to SAPF and IL-17A and IFN-γ elevation. Pulmonary samples of 2-month-old and 2-year-old mice were analyzed. (A) Representative micrographs of paraffin-embedded pulmonary samples stained immunohistochemically for p16 and p53 in bronchi and alveoli, with Hematoxylin staining the nuclei. (B) Percentage of p16- or p53-positive bronchial and alveolar cells. (C) Western blots of pulmonary extracts showing p16, p19, p53, SFTPC, α-SMA, Snail, type I collagen (collagen 1), IL-17A, IFNγ, TGF-β1, Smad2, p-Smad2(Ser465/467), p-Smad2/3(Ser423/425), Snail, IL-11, IL-11Rα1, MEK1/2, p-MEK1/2(Ser217/221), ERK1/2, and p-ERK1/2 (Thr202/Tyr204). β-actin was used as the loading control. (D) Protein levels relative to β-actin were assessed by densitometric analysis and normalized to the 2-month-old WT group. (E) Representative micrographs of paraffin-embedded pulmonary samples stained immunohistochemically for IL-17A in bronchi and alveoli, with hematoxylin staining for the nuclei. (F) Percentage of IL-17A-positive bronchial and alveolar cells. Three biological replicates were used per experiment. Values are the means ± SEM of three determinations per group. *$p < 0.05$; **$p < 0.01$; ***$p < 0.001$ compared with the 2-month-old group. Statistical analysis was performed with Student's t-test.

© 2023. Chen H et al. Published online at http://www.aginganddisease.org/EN/10.14336/AD.2023.0320

SUPPLEMENTARY DATA

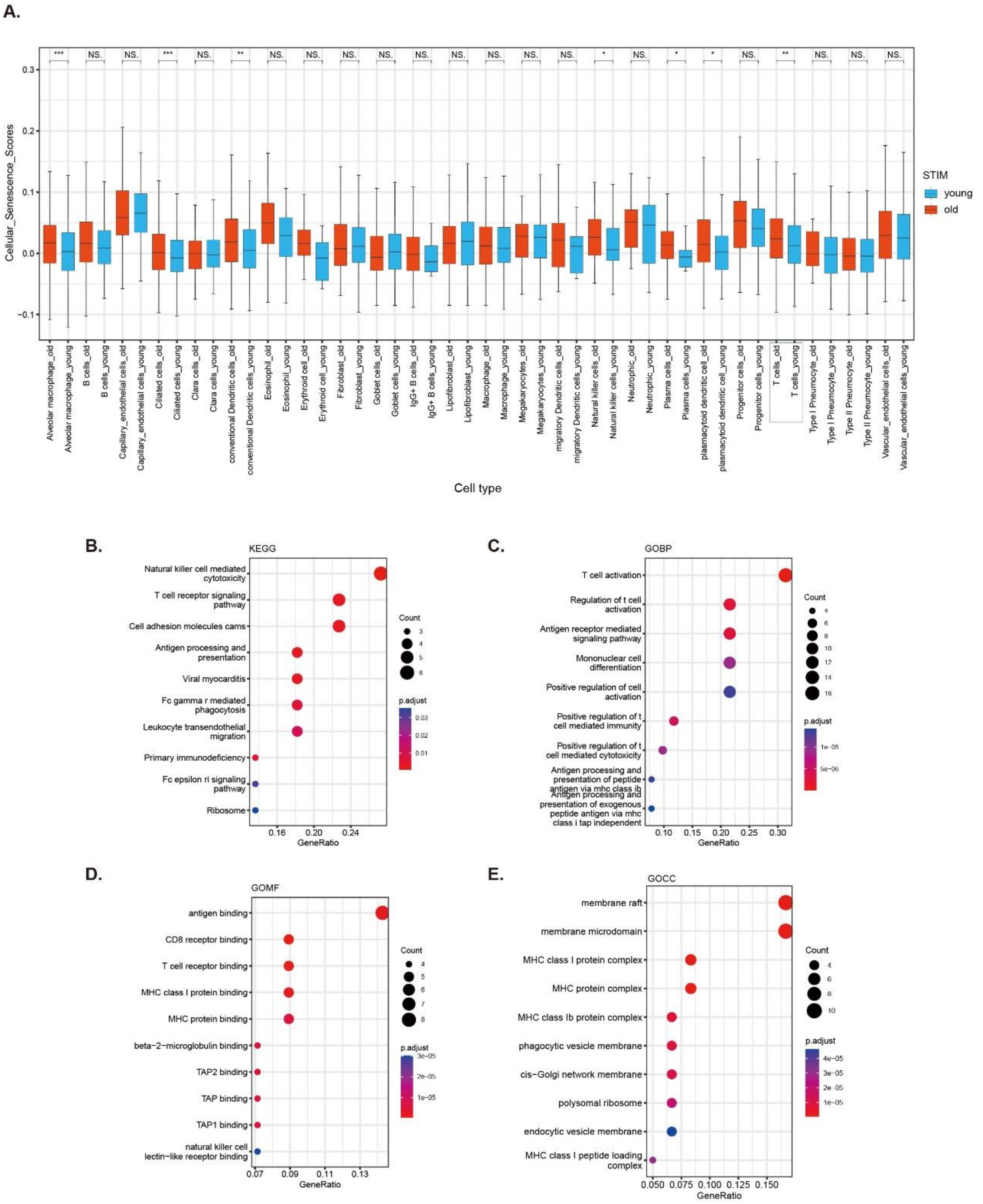

Supplementary Figure 3. Senescence and activation of T cells shown in physiologically aged lungs. (A) Gene set score analysis of cellular senescence in various lung cell types of different groups. (B-C) DEGs of young and aged T cells were used for KEGG enrichment analysis and GO-BP enrichment analysis. (D-E) Enrichment analysis revealed GO-MF and GO-CC analysis of young and aged lung T cells. Values are the mean ± SD. *p < 0.05; **p < 0.01; ***p <0.001 compared with 2-month-old WT group; ns, not significant (two-sided Wilcoxon rank-sum tests).

© 2023. Chen H et al. Published online at http://www.aginganddisease.org/EN/10.14336/AD.2023.0320

SUPPLEMENTARY DATA

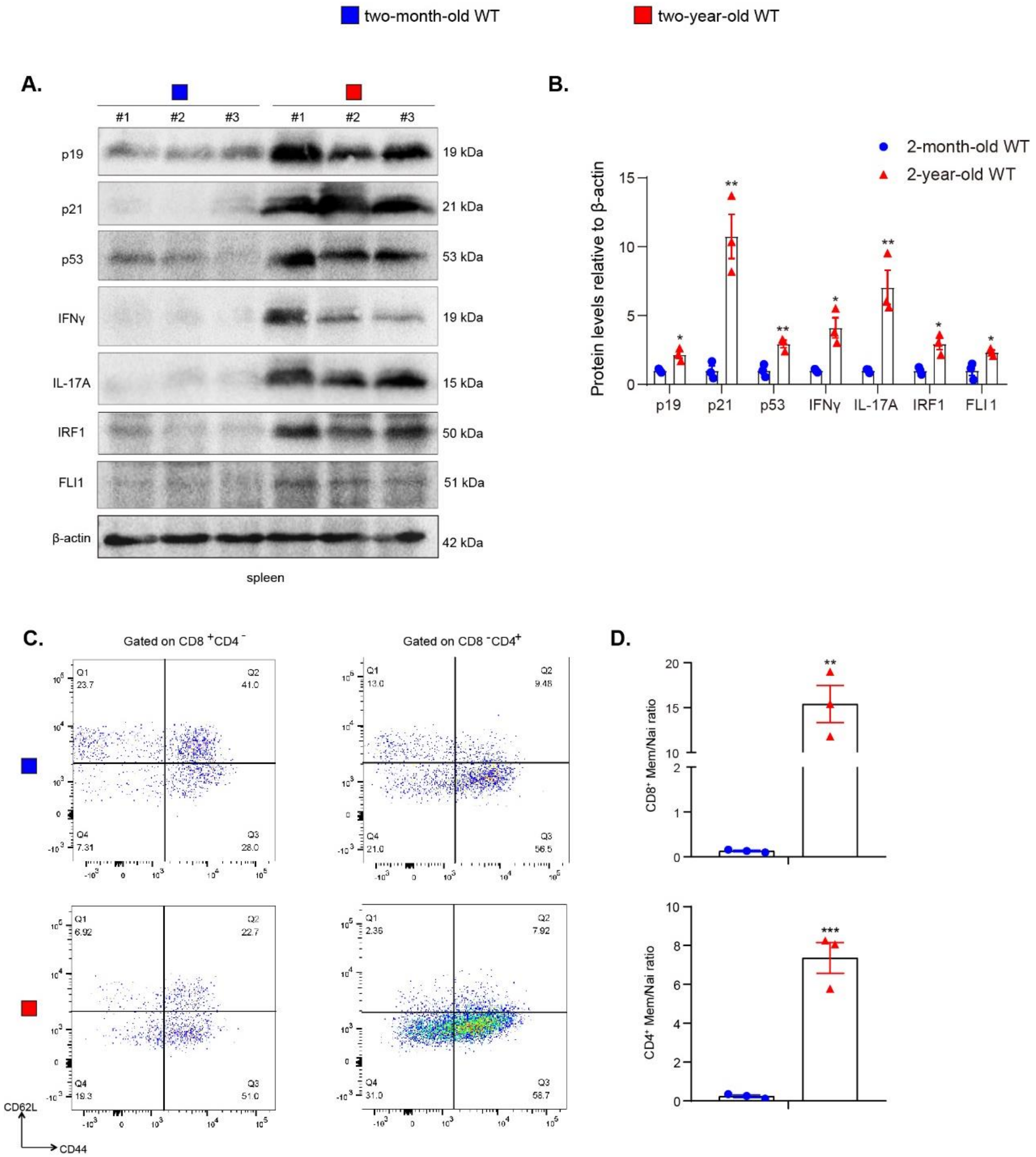

Supplementary Figure 4. Physiological aging increases IFNγ, IL-17A and IRF1 in splenic cells. Splenic samples of 2-month-old and 2-year-old mice were analyzed. (A) Western blots of splenic cell extracts showing p19, p21, p53, IFNγ, IL-17A, IRF1 and FLI1. β-actin was used as the loading control. (B) Protein levels relative to β-actin were assessed by densitometric analysis and normalized to the 2-month-old WT group. (C) Representative flow cytometric analyses of CD44 versus CD62L expression on $CD4^+$ T cell lineage or $CD8^+$ T cell lineage on total spleen cells from 2-month-old WT and 2-year-old WT mice. (D) The ratio of T_{EM} versus naïve T cells in the $CD4^+$ and $CD8^+$ compartments. Three biological replicates were used per experiment. Values are the means ± SEM of three determinations per group. $^{**}p < 0.01$; $^{***}p < 0.001$ compared with the 2-month-old group. Statistical analysis was performed with Student's t-test.

© 2023. Chen H et al. Published online at http://www.aginganddisease.org/EN/10.14336/AD.2023.0320

SUPPLEMENTARY DATA

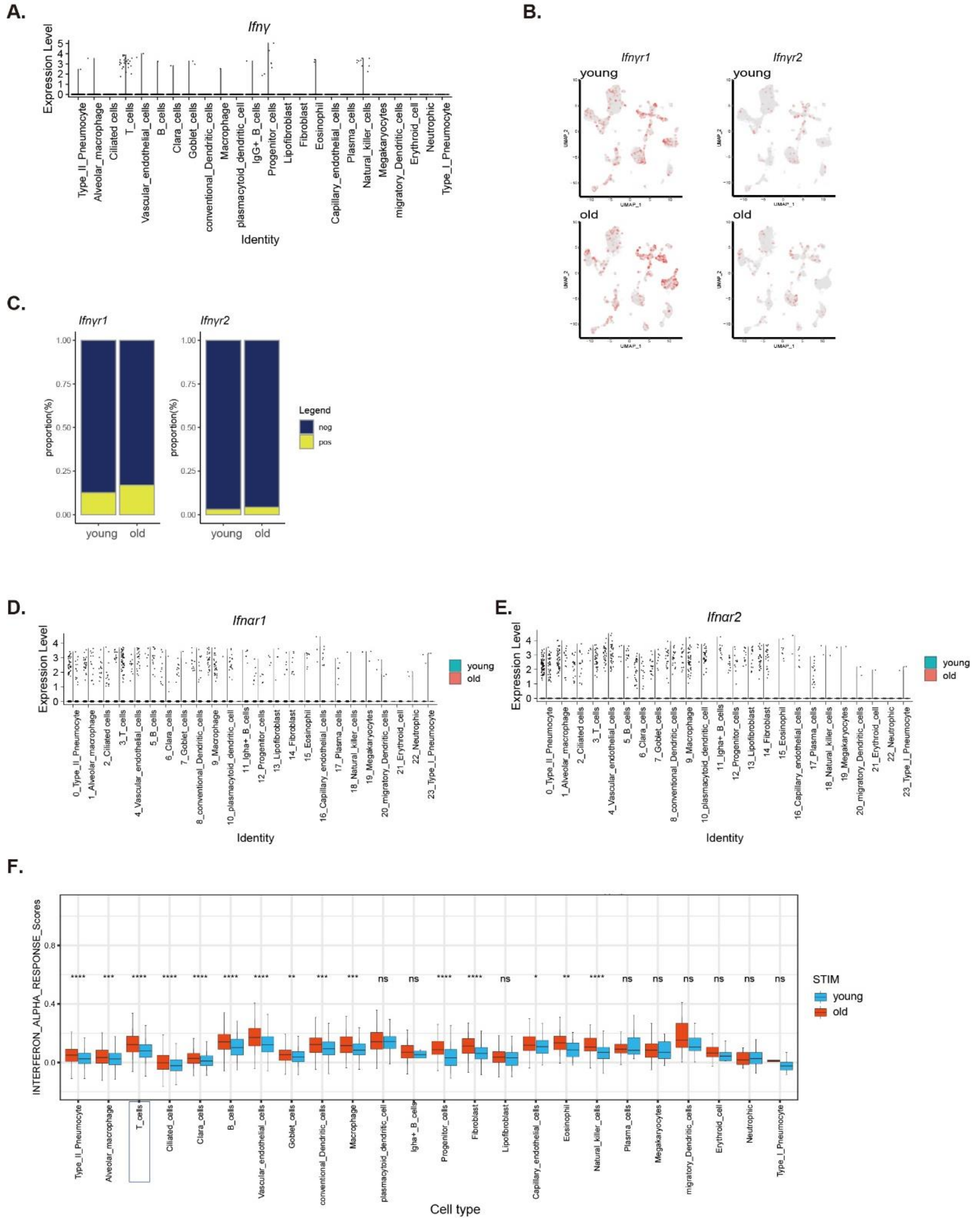

Supplementary Figure 5. Physiological aging increases responsiveness to IFNγ signaling in pulmonary cells. (A) *Ifnγ* expression was analyzed in single cells from different cell types. (B) UMAP plots showing the expression levels of *Ifnγr1* and *Ifnγr2* in 14,685 cells. (C) Percentage of cells expressing *Ifnγr1* and *Ifnγr2*. (D–E) Violin plots showing *Ifnαr1* and *Ifnαr2* expression in single cells from different cell types. (F) Combined log-normalized expression values of genes in response to IFNα in various cell types of the lung. Single cells were grouped by cell type and age. Values are the mean ± SD. *$p < 0.05$, **$p < 0.01$, ***$p < 0.001$, ****$p < 0.0001$ compared with the 2-month-old WT group; ns, not significant (two-sided Wilcoxon rank-sum tests).

© 2023. Chen H et al. Published online at http://www.aginganddisease.org/EN/10.14336/AD.2023.0320

SUPPLEMENTARY DATA

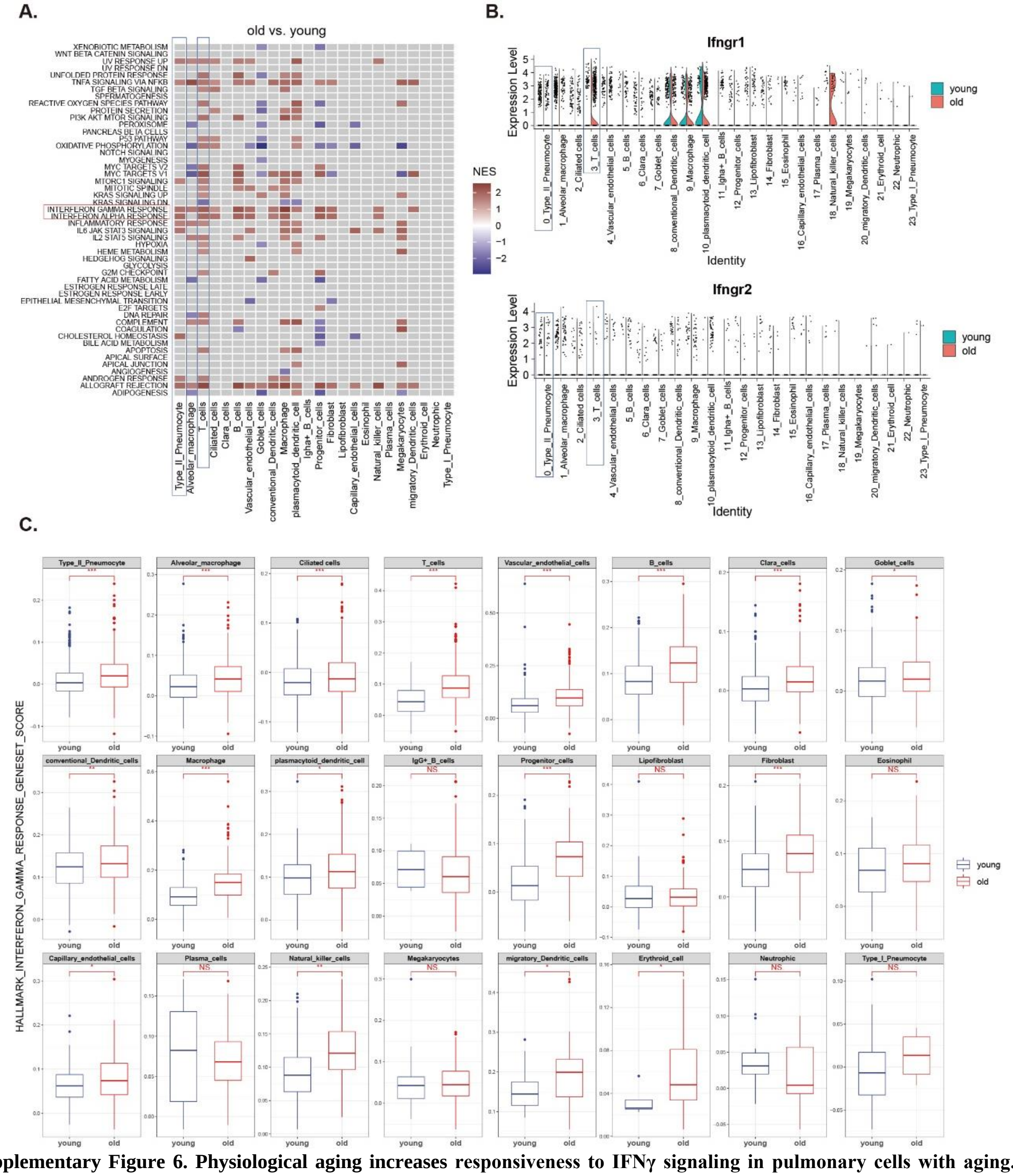

Supplementary Figure 6. Physiological aging increases responsiveness to IFNγ signaling in pulmonary cells with aging. (A) MSigDB Hallmarks (v.6.1) GSEA results for multiple cell types in the lung tissues from 2-month-old WT and 2-year-old WT mice. The normalized enrichment score is presented for each pathway with FDR < 0.05. (B) Violin plots showing *Ifnγr1* and *Ifnγr2* –which encode the IFNγ receptor expression by age and cell type. (C) Combined log-normalized expression values of genes in the IFNγ response in various cell types of the lung. Single cells were grouped by cell type and age. Expression values are represented as normalized log_2-transformed counts. Values are the mean ± SD. *p < 0.05, **p < 0.01, ***p < 0.001 compared with the 2-month-old group; ns, not significant (two-sided Wilcoxon rank-sum tests).

© 2023. Chen H et al. Published online at http://www.aginganddisease.org/EN/10.14336/AD.2023.0320

SUPPLEMENTARY DATA

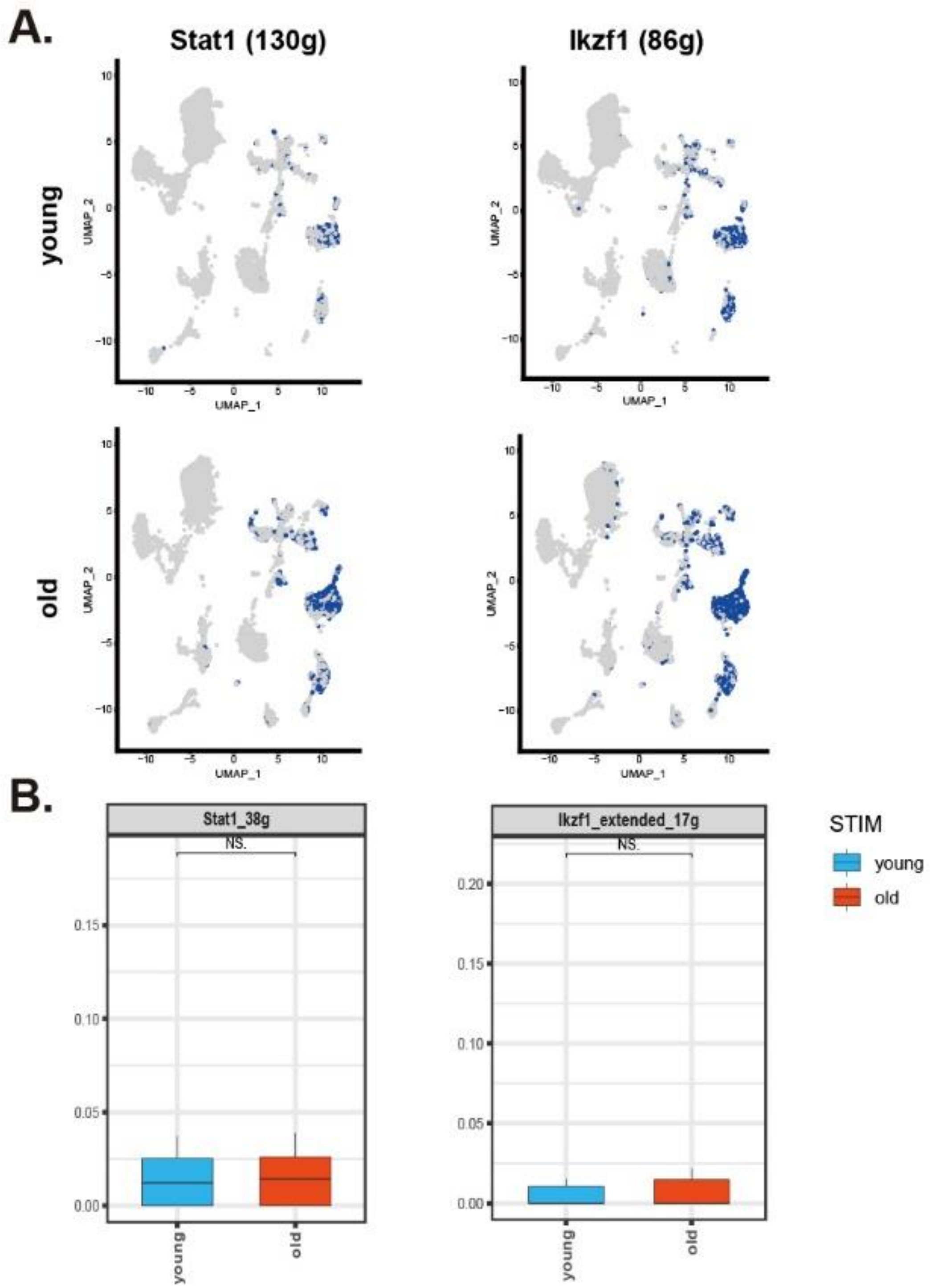

Supplementary Figure 7. Specific regulon activity of *Stat1* and *Ikzf1* in T cell subclusters, (A) Feature plots showing specific regulon activity (*Stat1* and *Ikzf1*) in pulmonary T cell subclusters between young and physiologically aged mice. Expression values are represented as normalized log_2-transformed counts. Values are the mean ± SD.

© 2023. Chen H et al. Published online at http://www.aginganddisease.org/EN/10.14336/AD.2023.0320

Figure S8

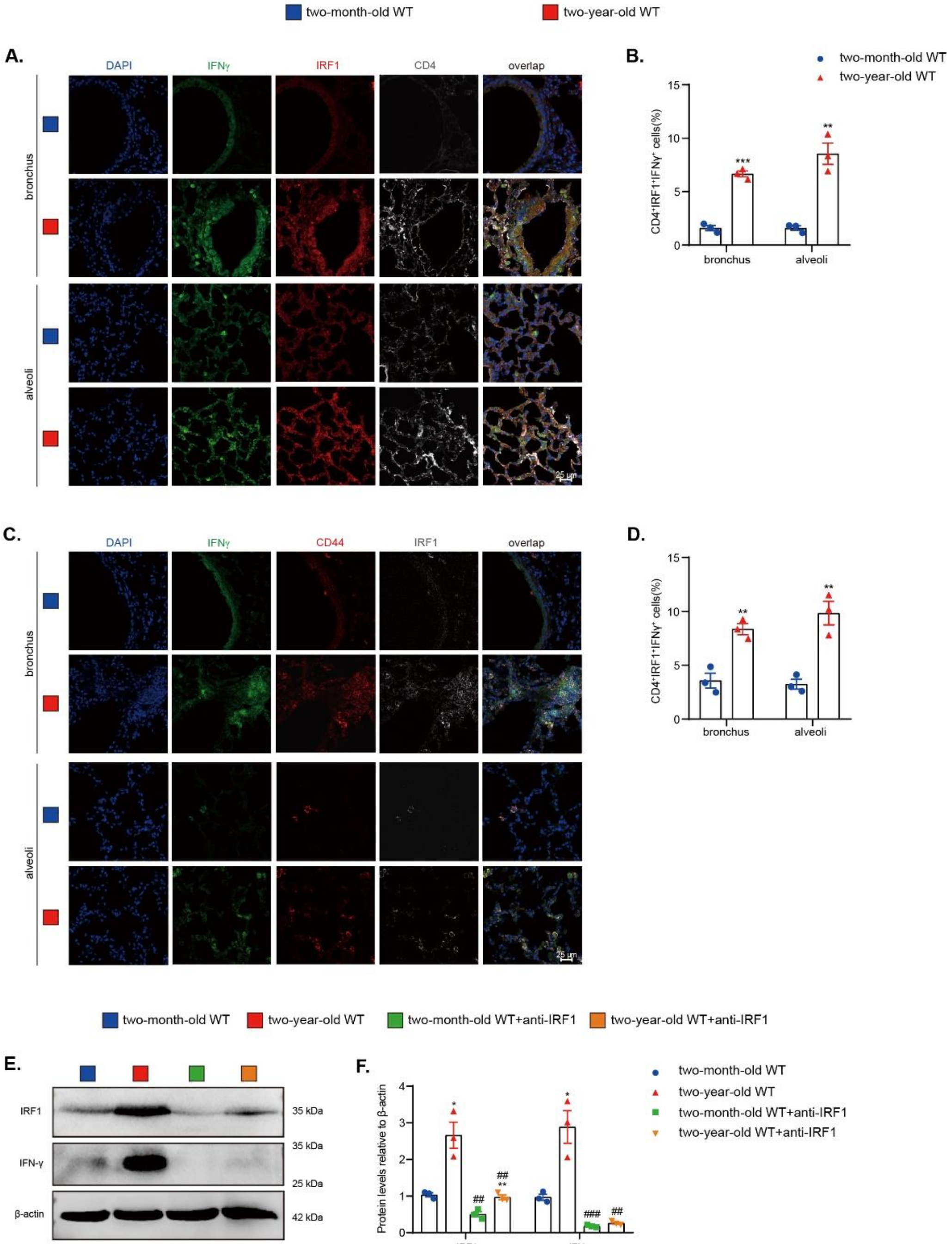

Supplementary Figure 8. Accumulated IRF1$^+$CD4$^+$ T_{EM} produces IFNγ in lungs during aging and anti-IRF1 primary antibody treatment inhibits the expression of IFNγ. (A) Representative micrographs of pulmonary samples immunofluorescently stained for IFNγ, IRF1, and CD4, with DAPI staining the nucleus. (B) The percentage of CD4-, IRF1-, and IFNγ-positive cells. (C) Representative micrographs of pulmonary samples immunofluorescently stained for IFNγ, IRF1, and CD44, with DAPI staining the nucleus. (D) The percentage of CD44-, IRF1-, and IFNγ-positive cells. Three biological replicates were used per experiment (N = 3). Values are the mean ± SEM of six determinations. **$p < 0.01$, ***$p < 0.001$ compared with the 2-month-old WT group. (E) Western blots showing IRF1 and IFNγ in pulmonary CD4$^+$ T_{EM} cells of 2-month-old WT and 2-year-old WT mice treated with or without anti-IRF1 antibody. β-actin was used as the loading control. (F) Protein levels relative to β-actin were assessed by densitometric analysis and normalized to the 2-month-old WT group. Three biological replicates were used per experiment (N = 3). Values are the mean ± SEM of six

© 2023. Chen H et al. Published online at http://www.aginganddisease.org/EN/10.14336/AD.2023.0320

determinations. *p < 0.05, **p < 0.01 compared with the 2-month-old WT group of the same treatment; ##p < 0.01, ###p < 0.001 compared with the untreated group of the same age.

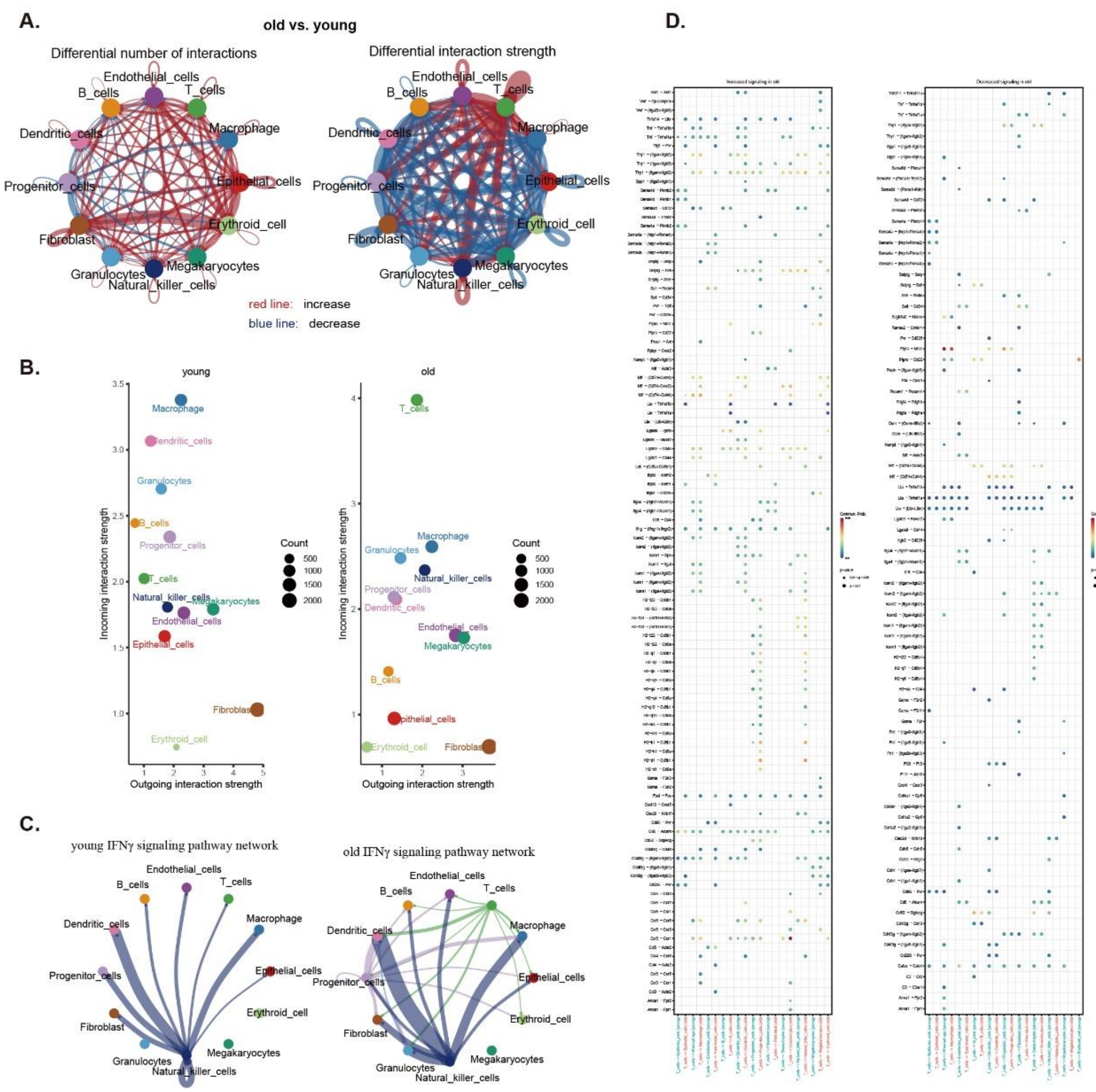

Supplementary Figure 9. Cell communication between pulmonary T cells and other surrounding cells increases with aging. (A) Signaling is expressed differentially when T cells function on other types of cells. (B) Dot plots revealed cell-cell communication between the young and old groups. The size of the dots represents the strength of the cell-cell communication, and the spectrum of color indicates different types of cells. (C–D) Heat map showing net outgoing and incoming signals in respective cell types as predicted by the CellChat algorithm. (E) IFNγ signaling from natural killer cells, T cells, and progenitor cells and towards their target cells as predicted using the CellChat algorithm.

© 2023. Chen H et al. Published online at http://www.aginganddisease.org/EN/10.14336/AD.2023.0320

SUPPLEMENTARY DATA

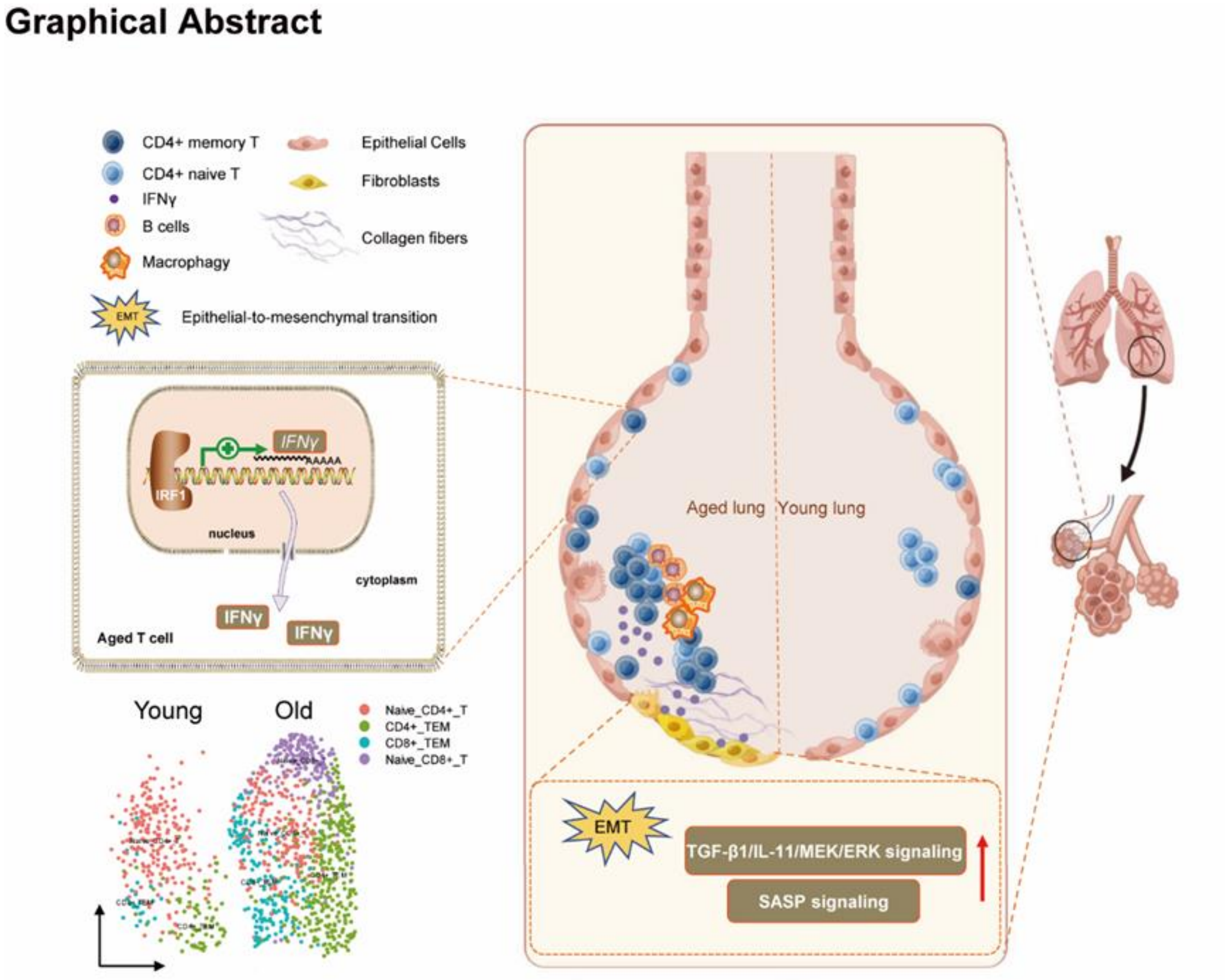

Graphical Abstract. Physiological aging increased pulmonary CD4$^+$ T$_{EM}$ cells to promote pathogenesis of senescence-associated pulmonary fibrosis (SAPF). *IFNγ* transcribed by IRF1 in CD4$^+$ T$_{EM}$ cells promoted epithelial-to-mesenchymal transition of alveolar type II epithelial cells by activating TGF-β1/IL-11/MEK/ERK signaling. The IFNγ produced by CD4$^+$ T$_{EM}$ cells in physiologically aged lung could be a therapeutic target for preventing SAPF.

Supplementary Table 1. The_complete_list_of_SASP_genes_used.

gene_symbol	Ifng	Ccna1	Mmp10
Il6	Mif	Ccna2	Mmp12
Il7	Areg	Cdc16	Mmp13
Il1a	Ereg	Cdc23	Mmp14
Il1b	Egf	Cdc26	Timp1
Il13	Fgf2	Cdc27	Ifng
Il15	Hgf	Cdk2	Mif
Cxcl	Fgf7	Cdk4	Areg
Ccl	Ang	Cdk6	Ereg
Il8	Cxcl12	Cdkn1a	Egf
Ccl8	Pigf	Cdkn1b	Fgf2
Mcpt4	Ngf	Cdkn2a	Hgf
Ccl3	Igfbp2	Cdkn2b	Fgf7
Ccl20	Igfbp3	Cdkn2c	Ang
Ccl11	Igfbp4	Cdkn2d	Cxcl12

© 2023. Chen H et al. Published online at http://www.aginganddisease.org/EN/10.14336/AD.2023.0320

Ccl25	Igfbp6	Cebpb	Pigf
Cxcl5	Igfbp7	Ehmt1	Ngf
Ccl1	Mmp1	Ehmt2	Igfbp2
Cxcl11	Mmp3	Fos	Igfbp3
Timp2	Mmp10	Fzr1	Igfbp4
Pai1	Mmp12	Igfbp7	Igfbp6
Pai2	Mmp13	Il1a	Igfbp7
Plat	Mmp14	Il6	Mmp1
Plau	Timp1	Jun	Mmp3
Ctsb	Ifng	Mapk1	Mmp10
Icam1	Mif	Mapk3	Mmp12
Icam3	Areg	Mapk7	Mmp13
Tnfrsf11b	Ereg	Nfkb1	Mmp14
Fas	Egf	Rela	Timp1
Plaur	Fgf2	Rps27a	Ifng
Il6st	Hgf	Rps6ka1	Mif
Egfr	Fgf7	Rps6ka2	Areg
Nos2	Ang	Rps6ka3	Ereg
Fn1	Cxcl12	Stat3	Egf
Anapc1	Pigf	Uba52	Fgf2
Anapc10	Ngf	Ubb	Hgf
Anapc11	Igfbp2	Ubc	Fgf7
Anapc15	Igfbp3	Ube2c	Ang
Anapc16	Igfbp4	Ube2d1	Cxcl12
Anapc2	Igfbp6	Ube2e1	Pigf
Anapc4	Igfbp7	Ube2s	Ngf
Anapc5	Mmp1	Ccna1	Mmp10
Anapc7	Mmp3	Ccna2	Mmp12

Supplementary Table 2. Differential_expressed_genes.

	p_val	avg_log2FC	pct.1	pct.2	p_val_adj	SYMBOL
Igha	0	3.314541	0.266	0.043	0	Igha
Ighm	0	3.765107	0.379	0.082	0	Ighm
Igkc	0	3.875735	0.54	0.11	0	Igkc
Malat1	0	1.091139	0.945	0.889	0	Malat1
Scgb1a1	0	-1.60536	0.939	0.998	0	Scgb1a1
Sftpa1	0	-1.28785	0.488	0.767	0	Sftpa1
Sftpb	0	-1.32174	0.39	0.705	0	Sftpb
Sftpc	0	-1.19561	0.844	0.963	0	Sftpc
Sftpd	1.40E-283	-1.30084	0.262	0.547	2.47E-279	Sftpd
Igj	2.01E-270	3.276419	0.216	0.029	3.55E-266	Igj
Lyz2	1.72E-261	-0.86851	0.721	0.89	3.04E-257	Lyz2
Cbr2	3.06E-257	-0.89924	0.398	0.681	5.41E-253	Cbr2
Atp1b1	2.54E-251	-1.39946	0.155	0.406	4.49E-247	Atp1b1
B2m	9.39E-235	0.822978	0.79	0.622	1.66E-230	B2m
Npc2	1.15E-223	-1.00722	0.437	0.666	2.04E-219	Npc2
H2-D1	2.19E-201	0.879352	0.717	0.569	3.87E-197	H2-D1
Hc	1.21E-194	-1.24763	0.147	0.367	2.14E-190	Hc
Slc34a2	3.07E-189	-0.93752	0.252	0.495	5.43E-185	Slc34a2
Sfta2	1.62E-188	-1.30324	0.114	0.314	2.86E-184	Sfta2
Wfdc2	4.10E-185	-0.9373	0.261	0.498	7.24E-181	Wfdc2
Cxcl15	3.19E-175	-0.79978	0.269	0.506	5.64E-171	Cxcl15
H2-K1	1.49E-173	1.046913	0.563	0.389	2.63E-169	H2-K1
Selenbp1	2.62E-170	-1.05394	0.16	0.364	4.64E-166	Selenbp1
Rnase4	2.66E-161	-1.08789	0.182	0.383	4.70E-157	Rnase4
S100g	9.91E-157	-1.21334	0.108	0.285	1.75E-152	S100g
Cd52	1.71E-153	1.103288	0.353	0.166	3.03E-149	Cd52
Gm26924	1.85E-150	1.474313	0.449	0.276	3.28E-146	Gm26924
Lyz1	1.23E-146	-1.82116	0.105	0.272	2.18E-142	Lyz1
Chi3l1	1.99E-142	-0.8365	0.217	0.419	3.52E-138	Chi3l1
H2-Q7	3.52E-141	1.313234	0.175	0.044	6.22E-137	H2-Q7

© 2023. Chen H et al. Published online at http://www.aginganddisease.org/EN/10.14336/AD.2023.0320

SUPPLEMENTARY DATA

Ccl5	1.37E-140	1.960557	0.192	0.057	2.43E-136	Ccl5
Lamp3	5.53E-135	-0.97131	0.156	0.336	9.78E-131	Lamp3
mt-Nd2	3.95E-129	-0.64705	0.509	0.678	6.99E-125	mt-Nd2
Scgb3a2	7.83E-124	-1.01937	0.144	0.31	1.38E-119	Scgb3a2
Cldn18	1.26E-123	-0.9975	0.093	0.242	2.22E-119	Cldn18
Ager	1.86E-122	-0.99672	0.101	0.254	3.30E-118	Ager
Napsa	3.36E-121	-0.91098	0.215	0.39	5.94E-117	Napsa
Ctsh	1.08E-120	-0.8805	0.208	0.381	1.90E-116	Ctsh
Dram1	2.64E-118	-0.96131	0.104	0.253	4.66E-114	Dram1
Prdx6	4.88E-112	-0.88788	0.199	0.364	8.63E-108	Prdx6
Actb	8.42E-111	0.532219	0.887	0.838	1.49E-106	Actb
Mgst1	1.77E-108	-0.67577	0.207	0.383	3.14E-104	Mgst1
Rac2	2.72E-107	1.182581	0.173	0.058	4.81E-103	Rac2
Srgn	4.15E-105	0.941537	0.29	0.142	7.34E-101	Srgn
App	4.25E-103	-0.65435	0.253	0.426	7.50E-99	App
mt-Rnr1	9.06E-100	1.253979	0.345	0.208	1.60E-95	mt-Rnr1
Wbp5	2.31E-99	-0.87061	0.129	0.27	4.09E-95	Wbp5
Ppp1r14c	3.10E-98	-0.98643	0.05	0.157	5.48E-94	Ppp1r14c
Wfdc17	2.64E-95	1.715717	0.139	0.043	4.66E-91	Wfdc17
Ear2	2.59E-92	-1.356	0.063	0.172	4.57E-88	Ear2
Cyp2f2	3.73E-91	-0.88837	0.194	0.344	6.59E-87	Cyp2f2
Chchd10	6.61E-89	-0.70627	0.118	0.251	1.17E-84	Chchd10
S100a8	6.00E-87	1.651056	0.162	0.062	1.06E-82	S100a8
H2-Q6	9.23E-85	1.006713	0.105	0.026	1.63E-80	H2-Q6
Junb	4.47E-84	1.06912	0.273	0.149	7.90E-80	Junb
S100a9	1.80E-83	1.69401	0.134	0.045	3.18E-79	S100a9
Iglc2	5.68E-83	1.21371	0.1	0.024	1.00E-78	Iglc2
Ptprcap	1.17E-82	1.045816	0.111	0.03	2.07E-78	Ptprcap
Bex4	2.11E-81	-0.87394	0.032	0.117	3.72E-77	Bex4
Rgcc	3.14E-81	-0.76093	0.101	0.221	5.55E-77	Rgcc
Ptprc	3.19E-81	0.90499	0.27	0.147	5.64E-77	Ptprc
Cd36	6.35E-81	-0.42261	0.264	0.432	1.12E-76	Cd36
Psmb8	1.43E-78	0.955323	0.201	0.093	2.53E-74	Psmb8
Ms4a4b	3.86E-78	1.306984	0.118	0.036	6.82E-74	Ms4a4b
Rpl13a	4.31E-78	0.69322	0.66	0.607	7.62E-74	Rpl13a
Lpcat1	4.81E-78	-0.69354	0.131	0.258	8.50E-74	Lpcat1
mt-Nd5	4.98E-78	-0.48631	0.468	0.618	8.81E-74	mt-Nd5
Ces1d	5.32E-75	-0.56471	0.159	0.294	9.40E-71	Ces1d
Muc1	5.54E-75	-0.6834	0.071	0.172	9.79E-71	Muc1
Ms4a6b	1.64E-74	0.939152	0.113	0.034	2.90E-70	Ms4a6b
Egfl6	1.70E-73	-0.78978	0.052	0.142	3.01E-69	Egfl6
Plac8	3.47E-73	1.176983	0.12	0.039	6.13E-69	Plac8
Lgi3	4.87E-72	-0.74624	0.073	0.173	8.61E-68	Lgi3
Gimap4	6.57E-71	0.918014	0.103	0.03	1.16E-66	Gimap4
Arhgdib	5.76E-70	0.825002	0.238	0.127	1.02E-65	Arhgdib
Errfi1	1.41E-69	-0.79228	0.058	0.148	2.50E-65	Errfi1
Dstn	3.11E-69	-0.65932	0.14	0.259	5.49E-65	Dstn
H2-T23	2.08E-68	0.898791	0.188	0.09	3.67E-64	H2-T23
Rps9	4.88E-67	0.575459	0.657	0.6	8.63E-63	Rps9
Fyb	9.19E-66	0.984208	0.158	0.069	1.62E-61	Fyb
S100a6	2.27E-62	0.837445	0.257	0.151	4.01E-58	S100a6
Dbi	8.41E-62	-0.50106	0.225	0.357	1.49E-57	Dbi
mt-Nd4	1.31E-61	-0.35344	0.536	0.668	2.32E-57	mt-Nd4
H2afj	9.54E-61	-0.59791	0.149	0.261	1.69E-56	H2afj
Pabpc1	2.26E-59	0.654306	0.484	0.388	3.99E-55	Pabpc1
Aox3	1.23E-58	-0.73131	0.049	0.125	2.17E-54	Aox3
Cd97	1.56E-58	0.888523	0.13	0.054	2.76E-54	Cd97
Sparc	4.88E-58	-0.73073	0.07	0.157	8.63E-54	Sparc
Il2rg	7.87E-58	0.800546	0.121	0.048	1.39E-53	Il2rg
Fasn	4.24E-57	-0.52102	0.105	0.205	7.49E-53	Fasn
Socs2	1.53E-55	-0.62448	0.099	0.193	2.70E-51	Socs2
Alcam	1.54E-55	-0.5393	0.194	0.311	2.73E-51	Alcam
Sp100	2.38E-55	0.790573	0.106	0.039	4.20E-51	Sp100
mt-Nd1	5.02E-55	-0.31795	0.638	0.751	8.87E-51	mt-Nd1
Coro1a	2.45E-54	0.809759	0.12	0.049	4.33E-50	Coro1a
Ets1	6.73E-54	0.868418	0.115	0.046	1.19E-49	Ets1
Mettl7a1	2.56E-52	-0.53106	0.137	0.239	4.52E-48	Mettl7a1
Ptpn18	7.92E-51	0.779688	0.119	0.05	1.40E-46	Ptpn18
Ly6c2	1.33E-50	1.107827	0.104	0.041	2.36E-46	Ly6c2
Prnp	2.90E-50	-0.60262	0.086	0.169	5.12E-46	Prnp

© 2023. Chen H et al. Published online at http://www.aginganddisease.org/EN/10.14336/AD.2023.0320

Zdhhc3	3.87E-50	-0.68582	0.07	0.148	6.83E-46	Zdhhc3
Klf6	1.88E-49	0.773729	0.229	0.139	3.32E-45	Klf6
Stk17b	5.69E-48	0.770126	0.102	0.041	1.01E-43	Stk17b
Mt1	8.59E-48	-0.57649	0.165	0.268	1.52E-43	Mt1
Gde1	1.03E-47	-0.54943	0.072	0.149	1.82E-43	Gde1
Mndal	3.97E-47	0.727721	0.123	0.055	7.02E-43	Mndal
Aplp2	6.42E-46	-0.46069	0.199	0.307	1.13E-41	Aplp2
Abca3	9.84E-46	-0.49957	0.101	0.187	1.74E-41	Abca3
Cldn3	1.42E-45	-0.46186	0.109	0.196	2.50E-41	Cldn3
Pfn1	3.29E-45	0.616972	0.421	0.331	5.81E-41	Pfn1
Arl6ip1	4.15E-45	-0.47254	0.261	0.37	7.33E-41	Arl6ip1
Crip1	4.44E-44	0.373519	0.442	0.343	7.85E-40	Crip1
H2-Eb1	7.94E-44	0.729963	0.341	0.251	1.40E-39	H2-Eb1
Abcd3	8.97E-44	-0.57178	0.068	0.139	1.59E-39	Abcd3
Cpm	1.34E-42	-0.57459	0.076	0.149	2.37E-38	Cpm
Sdc4	1.51E-42	-0.50582	0.132	0.22	2.66E-38	Sdc4
Psmb9	2.83E-42	0.673974	0.11	0.049	5.00E-38	Psmb9
Ptprf	3.72E-42	-0.50995	0.091	0.169	6.57E-38	Ptprf
Cd9	1.49E-41	-0.48075	0.331	0.443	2.64E-37	Cd9
Lsp1	1.71E-41	0.695948	0.209	0.128	3.03E-37	Lsp1
Pi4k2b	2.29E-41	-0.5737	0.048	0.108	4.04E-37	Pi4k2b
Pdcd4	2.35E-41	0.733655	0.129	0.064	4.16E-37	Pdcd4
Samhd1	2.49E-41	0.803362	0.143	0.075	4.41E-37	Samhd1
Prr15l	1.96E-40	-0.51015	0.079	0.15	3.46E-36	Prr15l
Eef1a1	2.00E-40	0.464926	0.622	0.591	3.53E-36	Eef1a1
Hp	4.11E-40	-0.36406	0.249	0.358	7.26E-36	Hp
Tmsb10	4.41E-40	0.478985	0.46	0.37	7.79E-36	Tmsb10
Pon3	8.40E-39	-0.5387	0.059	0.121	1.48E-34	Pon3
Shisa5	2.57E-38	0.679769	0.209	0.132	4.54E-34	Shisa5
Btg1	1.68E-37	0.714805	0.145	0.08	2.96E-33	Btg1
Kcnj15	1.84E-37	-0.51505	0.048	0.105	3.24E-33	Kcnj15
Selplg	1.44E-36	0.703554	0.116	0.057	2.55E-32	Selplg
Krt19	2.33E-36	-0.49693	0.056	0.115	4.13E-32	Krt19
Rpsa-ps10	2.47E-36	0.652832	0.295	0.216	4.36E-32	Rpsa-ps10
Atp8a1	4.80E-36	-0.43368	0.115	0.192	8.48E-32	Atp8a1
Mrc1	5.40E-36	-0.69785	0.08	0.147	9.54E-32	Mrc1
Bsg	7.28E-36	-0.36557	0.178	0.268	1.29E-31	Bsg
Il33	7.71E-36	-0.49787	0.056	0.115	1.36E-31	Il33
Por	1.27E-35	-0.47473	0.097	0.169	2.25E-31	Por
Carkd	2.53E-35	-0.49861	0.063	0.124	4.48E-31	Carkd
Actr3	2.65E-35	0.61075	0.212	0.137	4.69E-31	Actr3
Ly6a	8.24E-35	0.548655	0.218	0.14	1.46E-30	Ly6a
Marcks	2.18E-34	0.64227	0.106	0.052	3.86E-30	Marcks
Rps18	2.35E-34	0.577957	0.555	0.52	4.15E-30	Rps18
Ier2	3.06E-34	0.677001	0.197	0.126	5.41E-30	Ier2
Nfkbia	3.52E-34	0.601074	0.261	0.182	6.22E-30	Nfkbia
Idh2	5.19E-34	-0.44397	0.052	0.108	9.18E-30	Idh2
Sdc1	6.30E-34	-0.48822	0.066	0.125	1.11E-29	Sdc1
Atp6v0d2	6.88E-34	-0.57459	0.062	0.122	1.22E-29	Atp6v0d2
AW112010	1.12E-33	0.601377	0.14	0.078	1.98E-29	AW112010
Scp2	1.55E-33	-0.37137	0.175	0.261	2.74E-29	Scp2
Tpm1	1.92E-33	-0.43666	0.123	0.199	3.40E-29	Tpm1
Celf2	1.94E-33	0.710436	0.131	0.072	3.43E-29	Celf2
Iah1	2.07E-33	-0.48464	0.054	0.109	3.66E-29	Iah1
Prdx1	3.26E-33	-0.32245	0.342	0.447	5.77E-29	Prdx1
Elovl1	3.61E-33	-0.39685	0.128	0.206	6.38E-29	Elovl1
Rpsa	4.68E-33	0.612655	0.362	0.29	8.26E-29	Rpsa
Atrx	9.25E-33	0.583914	0.231	0.157	1.64E-28	Atrx
Msn	1.53E-32	0.61009	0.248	0.173	2.71E-28	Msn
Glul	8.48E-32	-0.41604	0.135	0.211	1.50E-27	Glul
Wdr89	1.03E-31	0.490595	0.529	0.48	1.83E-27	Wdr89
Ctsc	1.75E-31	-0.33834	0.179	0.264	3.09E-27	Ctsc
Gas6	2.04E-31	-0.41417	0.064	0.122	3.60E-27	Gas6
Atp6v1c2	2.47E-31	-0.49014	0.053	0.105	4.37E-27	Atp6v1c2
Secisbp2l	3.64E-31	-0.46982	0.074	0.135	6.44E-27	Secisbp2l
Rpl18	1.33E-30	0.61576	0.387	0.323	2.35E-26	Rpl18
Rps5	2.32E-30	0.521153	0.559	0.529	4.11E-26	Rps5
Ptpn6	2.91E-30	0.645587	0.13	0.073	5.14E-26	Ptpn6
Rps28	3.92E-30	-0.41205	0.367	0.465	6.93E-26	Rps28
Gm9843	5.49E-30	0.399763	0.594	0.558	9.71E-26	Gm9843

© 2023. Chen H et al. Published online at http://www.aginganddisease.org/EN/10.14336/AD.2023.0320

SUPPLEMENTARY DATA

Cd53	8.36E-30	0.542156	0.16	0.098	1.48E-25	Cd53
Tgoln1	9.97E-30	-0.36361	0.146	0.221	1.76E-25	Tgoln1
Arhgap30	1.57E-29	0.533432	0.119	0.065	2.77E-25	Arhgap30
Rplp2	1.75E-29	0.493134	0.506	0.456	3.09E-25	Rplp2
H2-Ab1	2.16E-29	0.500641	0.427	0.366	3.81E-25	H2-Ab1
Dcxr	5.26E-29	-0.41549	0.074	0.131	9.30E-25	Dcxr
Rps24	1.25E-28	0.500794	0.506	0.463	2.22E-24	Rps24
Ldhb	1.26E-28	-0.36113	0.091	0.153	2.23E-24	Ldhb
Phldb2	1.51E-28	-0.44874	0.068	0.123	2.66E-24	Phldb2
Emp3	2.15E-28	0.661058	0.143	0.086	3.80E-24	Emp3
Lcp1	3.91E-28	0.595605	0.262	0.193	6.91E-24	Lcp1
Car8	4.02E-28	-0.48154	0.073	0.128	7.10E-24	Car8
1600029D21Rik	6.02E-28	-0.56472	0.069	0.123	1.06E-23	1600029D21Rik
Psap	6.82E-28	0.614173	0.315	0.243	1.21E-23	Psap
Vamp8	9.37E-28	-0.25489	0.259	0.352	1.66E-23	Vamp8
Tspan13	3.10E-27	0.605662	0.114	0.063	5.48E-23	Tspan13
Lpl	3.81E-27	-0.37633	0.117	0.184	6.74E-23	Lpl
Gsta4	6.98E-27	-0.37754	0.053	0.102	1.23E-22	Gsta4
Trf	7.08E-27	-0.36236	0.125	0.193	1.25E-22	Trf
Suclg1	8.40E-27	-0.47553	0.076	0.13	1.49E-22	Suclg1
Neat1	1.38E-26	-0.25861	0.219	0.305	2.44E-22	Neat1
Slc12a2	3.24E-26	-0.41081	0.061	0.111	5.72E-22	Slc12a2
Nceh1	7.26E-26	-0.41322	0.066	0.117	1.28E-21	Nceh1
Ndufc2	1.09E-25	-0.36058	0.167	0.238	1.93E-21	Ndufc2
Aldh1a1	1.14E-25	-0.47156	0.099	0.158	2.02E-21	Aldh1a1
Zfp36l1	1.17E-25	0.642113	0.228	0.166	2.07E-21	Zfp36l1
Lpin2	2.83E-25	-0.38586	0.095	0.154	5.00E-21	Lpin2
Mid1ip1	4.34E-25	-0.34076	0.118	0.182	7.68E-21	Mid1ip1
Nucb2	1.97E-24	-0.40165	0.076	0.128	3.48E-20	Nucb2
Lmo7	2.79E-24	-0.41797	0.066	0.115	4.92E-20	Lmo7
Cyb5r3	4.47E-24	-0.33025	0.152	0.22	7.91E-20	Cyb5r3
Creg1	4.79E-24	-0.26147	0.172	0.245	8.46E-20	Creg1
Cox6b1	5.64E-24	-0.28761	0.273	0.357	9.96E-20	Cox6b1
Ankrd11	5.87E-24	0.5512	0.131	0.081	1.04E-19	Ankrd11
Cd302	8.82E-24	-0.51261	0.074	0.124	1.56E-19	Cd302
Atxn10	1.26E-23	-0.38787	0.065	0.112	2.22E-19	Atxn10
Gclc	3.25E-23	-0.35211	0.081	0.133	5.74E-19	Gclc
Acsl4	3.52E-23	-0.31024	0.108	0.167	6.23E-19	Acsl4
Gnai2	3.90E-23	0.572683	0.197	0.14	6.90E-19	Gnai2
Hmha1	4.21E-23	0.579476	0.105	0.06	7.45E-19	Hmha1
Cox7a2	6.81E-23	-0.28594	0.203	0.276	1.20E-18	Cox7a2
Mcl1	1.53E-22	0.634118	0.148	0.097	2.70E-18	Mcl1
Atp11a	2.14E-22	-0.3417	0.072	0.121	3.78E-18	Atp11a
Hbb-bs	4.02E-22	3.00016	0.174	0.127	7.10E-18	Hbb-bs
Rps14	8.17E-22	0.30064	0.698	0.697	1.44E-17	Rps14
Serinc3	1.43E-21	0.463022	0.335	0.275	2.52E-17	Serinc3
Capzb	1.98E-21	0.533539	0.212	0.155	3.50E-17	Capzb
Rgs2	3.71E-21	0.544167	0.119	0.074	6.55E-17	Rgs2
Gnb2l1	4.19E-21	0.481288	0.352	0.294	7.41E-17	Gnb2l1
Rpl8	5.13E-21	0.445495	0.478	0.442	9.06E-17	Rpl8
Ptms	7.31E-21	-0.3544	0.078	0.126	1.29E-16	Ptms
Gm8730	1.16E-20	0.528623	0.344	0.292	2.05E-16	Gm8730
Slfn2	1.60E-20	0.478355	0.166	0.114	2.82E-16	Slfn2
Apbb1ip	1.88E-20	0.477142	0.122	0.077	3.33E-16	Apbb1ip
Ctla2a	2.14E-20	0.507657	0.12	0.075	3.79E-16	Ctla2a
Acsl5	2.62E-20	-0.28373	0.063	0.107	4.63E-16	Acsl5
Psme2b	2.88E-20	0.533278	0.172	0.121	5.08E-16	Psme2b
Mbnl1	3.63E-20	0.545934	0.199	0.145	6.42E-16	Mbnl1
Gmfg	4.97E-20	0.520866	0.11	0.068	8.79E-16	Gmfg
Exosc7	7.52E-20	-0.31534	0.062	0.105	1.33E-15	Exosc7
Cadm1	8.62E-20	-0.32647	0.059	0.1	1.52E-15	Cadm1
Elf1	1.21E-19	0.554717	0.126	0.081	2.14E-15	Elf1
Arpc2	1.23E-19	0.387326	0.41	0.357	2.17E-15	Arpc2
Eno1	1.47E-19	0.515113	0.111	0.069	2.59E-15	Eno1
Scgb3a1	2.15E-19	-0.65397	0.11	0.164	3.80E-15	Scgb3a1
Rpl13a-ps1	3.04E-19	0.510626	0.115	0.073	5.37E-15	Rpl13a-ps1
Cmtm8	3.24E-19	-0.32447	0.059	0.1	5.72E-15	Cmtm8
H2-Aa	7.28E-19	0.572891	0.403	0.364	1.29E-14	H2-Aa
Abcg1	1.08E-18	-0.49629	0.065	0.106	1.91E-14	Abcg1
Hmgn1	1.26E-18	-0.30143	0.141	0.198	2.23E-14	Hmgn1

© 2023. Chen H et al. Published online at http://www.aginganddisease.org/EN/10.14336/AD.2023.0320

SUPPLEMENTARY DATA

Acot7	1.45E-18	-0.28072	0.08	0.126	2.57E-14	Acot7
Ncl	1.84E-18	0.471564	0.317	0.264	3.26E-14	Ncl
Oat	2.17E-18	-0.27943	0.089	0.136	3.83E-14	Oat
Ppp1r12a	2.58E-18	0.422818	0.103	0.064	4.55E-14	Ppp1r12a
Itgb2	2.97E-18	0.501414	0.113	0.072	5.25E-14	Itgb2
Macf1	3.62E-18	0.555943	0.191	0.142	6.39E-14	Macf1
Ifitm3	4.02E-18	0.487669	0.206	0.154	7.11E-14	Ifitm3
Ramp1	4.48E-18	-0.32406	0.072	0.115	7.91E-14	Ramp1
Ywhaz	5.31E-18	0.501564	0.237	0.184	9.38E-14	Ywhaz
Nipbl	5.34E-18	0.407279	0.138	0.092	9.44E-14	Nipbl
Cybb	5.88E-18	0.567108	0.178	0.129	1.04E-13	Cybb
Kmt2a	6.44E-18	0.557166	0.102	0.063	1.14E-13	Kmt2a
Cotl1	7.32E-18	0.478183	0.138	0.094	1.29E-13	Cotl1
Gngt2	9.68E-18	0.633819	0.109	0.07	1.71E-13	Gngt2
Eef2	1.76E-17	0.43783	0.32	0.27	3.11E-13	Eef2
Cystm1	2.02E-17	-0.32474	0.075	0.116	3.58E-13	Cystm1
Rps3	2.11E-17	0.379209	0.593	0.592	3.73E-13	Rps3
Ank3	2.99E-17	-0.26137	0.062	0.102	5.28E-13	Ank3
Sub1	3.10E-17	0.489617	0.274	0.224	5.47E-13	Sub1
Psme2	3.71E-17	0.506103	0.151	0.106	6.56E-13	Psme2
Rps11	5.02E-17	0.413401	0.515	0.497	8.88E-13	Rps11
Gm10275	7.97E-17	0.53029	0.268	0.22	1.41E-12	Gm10275
Rplp0	8.54E-17	0.423311	0.507	0.488	1.51E-12	Rplp0
Tln1	8.80E-17	0.496303	0.159	0.113	1.56E-12	Tln1
Fkbp4	9.41E-17	-0.27568	0.077	0.119	1.66E-12	Fkbp4
Soat1	1.11E-16	-0.28531	0.079	0.121	1.96E-12	Soat1
Rpl32	1.52E-16	0.354638	0.605	0.602	2.68E-12	Rpl32
Cdh1	1.54E-16	-0.26067	0.071	0.112	2.72E-12	Cdh1
Rpl4	3.38E-16	0.389883	0.507	0.486	5.98E-12	Rpl4
Ppp1ca	3.66E-16	0.469169	0.18	0.134	6.47E-12	Ppp1ca
Ly6e	4.52E-16	0.355528	0.517	0.492	7.99E-12	Ly6e
Cap1	5.31E-16	0.479582	0.129	0.089	9.39E-12	Cap1
Rpl18a	6.99E-16	0.41186	0.571	0.568	1.24E-11	Rpl18a
Ddx21	8.31E-16	0.448669	0.104	0.067	1.47E-11	Ddx21
Ptp4a2	9.07E-16	0.414427	0.227	0.177	1.60E-11	Ptp4a2
Ahnak	1.76E-15	0.517327	0.22	0.173	3.11E-11	Ahnak
Kmt2e	2.30E-15	0.450284	0.17	0.125	4.06E-11	Kmt2e
Acadl	3.11E-15	-0.27686	0.073	0.112	5.49E-11	Acadl
Ddx6	3.45E-15	0.440343	0.165	0.121	6.10E-11	Ddx6
Rpl36	4.43E-15	0.489888	0.39	0.358	7.83E-11	Rpl36
Cd24a	5.06E-15	0.64614	0.189	0.147	8.95E-11	Cd24a
Cd74	7.03E-15	0.354743	0.552	0.518	1.24E-10	Cd74
Ifngr1	7.21E-15	0.460001	0.17	0.126	1.27E-10	Ifngr1
Zfp36	1.27E-14	0.530333	0.162	0.12	2.24E-10	Zfp36
Rps10-ps1	1.36E-14	0.440686	0.291	0.246	2.40E-10	Rps10-ps1
Fos	1.44E-14	0.502302	0.218	0.173	2.54E-10	Fos
Gm6472	1.48E-14	0.469219	0.413	0.387	2.61E-10	Gm6472
Bptf	2.04E-14	0.393667	0.102	0.067	3.61E-10	Bptf
Btg2	2.12E-14	0.4433	0.135	0.096	3.74E-10	Btg2
Rap1a	2.32E-14	0.396979	0.155	0.112	4.10E-10	Rap1a
Hn1	2.43E-14	0.440867	0.104	0.069	4.29E-10	Hn1
Cyba	4.23E-14	0.371361	0.278	0.228	7.49E-10	Cyba
Arid4b	5.50E-14	0.439062	0.11	0.075	9.72E-10	Arid4b
Comt	5.84E-14	-0.27823	0.075	0.111	1.03E-09	Comt
Gm2a	9.35E-14	0.454667	0.122	0.086	1.65E-09	Gm2a
Arpc1b	1.01E-13	0.416726	0.223	0.178	1.78E-09	Arpc1b
Reep5	1.11E-13	-0.29794	0.126	0.17	1.97E-09	Reep5
Rock1	1.21E-13	0.410769	0.139	0.1	2.15E-09	Rock1
Ptbp3	1.41E-13	0.414398	0.143	0.104	2.50E-09	Ptbp3
Rpl14	1.72E-13	0.410939	0.428	0.401	3.05E-09	Rpl14
Tuba1a	7.23E-13	0.504514	0.159	0.121	1.28E-08	Tuba1a
Arid4a	9.05E-13	0.377809	0.101	0.068	1.60E-08	Arid4a
Myh9	9.74E-13	0.417314	0.174	0.134	1.72E-08	Myh9
Zbtb20	2.12E-12	0.531468	0.129	0.094	3.75E-08	Zbtb20
Smc4	2.63E-12	0.460298	0.126	0.091	4.65E-08	Smc4
Lrrc58	2.66E-12	0.296046	0.563	0.553	4.70E-08	Lrrc58
Tsc22d4	3.13E-12	0.409342	0.109	0.076	5.53E-08	Tsc22d4
Add3	4.16E-12	0.331134	0.129	0.093	7.36E-08	Add3
2410006H16Rik	5.15E-12	0.44394	0.111	0.078	9.10E-08	2410006H16Rik
Ankrd12	5.53E-12	0.45267	0.102	0.071	9.77E-08	Ankrd12

© 2023. Chen H et al. Published online at http://www.aginganddisease.org/EN/10.14336/AD.2023.0320

Jak1	6.01E-12	0.369944	0.144	0.107	1.06E-07	Jak1
Zc3h13	8.29E-12	0.447094	0.101	0.07	1.47E-07	Zc3h13
Nedd4	1.83E-11	-0.25827	0.094	0.13	3.23E-07	Nedd4
Rps10	2.27E-11	0.378378	0.309	0.271	4.00E-07	Rps10
Cdc42ep3	2.37E-11	-0.27186	0.088	0.123	4.18E-07	Cdc42ep3
Nktr	2.63E-11	0.381596	0.111	0.079	4.65E-07	Nktr
Hnrnpf	2.64E-11	0.404323	0.16	0.124	4.67E-07	Hnrnpf
Net1	3.51E-11	-0.2537	0.075	0.107	6.21E-07	Net1
Retnla	4.47E-11	-0.58881	0.082	0.117	7.91E-07	Retnla
Akap13	6.52E-11	0.423483	0.155	0.12	1.15E-06	Akap13
Actr2	7.46E-11	0.38631	0.176	0.138	1.32E-06	Actr2
Prkar1a	8.26E-11	0.399481	0.125	0.093	1.46E-06	Prkar1a
Prr13	1.06E-10	0.419239	0.174	0.138	1.87E-06	Prr13
Laptm5	1.31E-10	0.254162	0.267	0.222	2.32E-06	Laptm5
Tpr	1.40E-10	0.301167	0.176	0.139	2.48E-06	Tpr
Rps23	1.45E-10	0.401947	0.21	0.173	2.57E-06	Rps23
Eif3a	1.86E-10	0.39945	0.234	0.196	3.29E-06	Eif3a
Plek	1.88E-10	0.373347	0.118	0.086	3.32E-06	Plek
Ddx5	2.16E-10	0.291444	0.496	0.478	3.82E-06	Ddx5
Npm1	3.60E-10	0.384713	0.242	0.206	6.37E-06	Npm1
Rpl23a-ps3	3.73E-10	0.383123	0.298	0.264	6.59E-06	Rpl23a-ps3
Dazap2	4.05E-10	0.412148	0.155	0.122	7.15E-06	Dazap2
Vasp	4.25E-10	0.41059	0.1	0.072	7.50E-06	Vasp
Rpl13	6.21E-10	0.405229	0.274	0.241	1.10E-05	Rpl13
Clk1	7.51E-10	0.401128	0.106	0.078	1.33E-05	Clk1
Rps15	1.33E-09	0.34754	0.478	0.468	2.35E-05	Rps15
Tapbp	1.34E-09	0.384716	0.133	0.102	2.37E-05	Tapbp
Wnk1	1.46E-09	0.313263	0.103	0.075	2.59E-05	Wnk1
Brd2	2.12E-09	0.33245	0.131	0.1	3.75E-05	Brd2
Cd47	2.99E-09	0.272939	0.267	0.228	5.29E-05	Cd47
Gm10269	4.20E-09	0.304455	0.432	0.414	7.43E-05	Gm10269
Pkm	5.43E-09	0.422756	0.144	0.114	9.61E-05	Pkm
Tmem50a	6.16E-09	0.313631	0.229	0.194	0.000109	Tmem50a
Rps19	9.46E-09	0.274656	0.672	0.694	0.000167	Rps19
Gm7808	1.73E-08	0.377093	0.273	0.244	0.000306	Gm7808
Foxp1	1.93E-08	0.398159	0.156	0.126	0.000342	Foxp1
Ythdc1	2.58E-08	0.351939	0.1	0.075	0.000456	Ythdc1
Rbm39	3.17E-08	0.285872	0.318	0.286	0.00056	Rbm39
Rps3a2	3.62E-08	0.292355	0.258	0.225	0.000639	Rps3a2
Gm9493	5.14E-08	0.383851	0.316	0.292	0.000908	Gm9493
Atf4	8.68E-08	0.355056	0.107	0.082	0.001534	Atf4
Ptges3	1.58E-07	0.384683	0.128	0.102	0.002801	Ptges3
Tubb5	1.95E-07	0.313142	0.136	0.109	0.003452	Tubb5
Srsf2	2.37E-07	0.285762	0.109	0.084	0.004183	Srsf2
Eef1g	3.14E-07	0.367003	0.21	0.182	0.005549	Eef1g
Txnip	3.39E-07	0.350914	0.205	0.177	0.005993	Txnip
Ubc	3.45E-07	0.302143	0.321	0.295	0.0061	Ubc
Rps8	4.75E-07	0.27853	0.275	0.246	0.008404	Rps8
Rps16	5.37E-07	0.351584	0.198	0.17	0.009498	Rps16
G3bp2	6.08E-07	0.291481	0.117	0.092	0.010742	G3bp2
Stat3	6.40E-07	0.366042	0.1	0.077	0.011312	Stat3
Ube2d3	6.85E-07	0.325573	0.147	0.121	0.012116	Ube2d3
Gm10116	7.35E-07	0.282204	0.507	0.491	0.012989	Gm10116
Rps20	8.16E-07	0.28427	0.408	0.391	0.014429	Rps20
Ptpn1	8.49E-07	0.364638	0.108	0.084	0.015006	Ptpn1
Sfpq	1.07E-06	0.295874	0.112	0.089	0.018827	Sfpq
Slc3a2	1.21E-06	0.295767	0.1	0.078	0.021301	Slc3a2
Rbm5	1.21E-06	0.328253	0.11	0.087	0.021323	Rbm5
Gm11808	1.23E-06	0.287214	0.216	0.189	0.021692	Gm11808
Prrc2c	1.26E-06	0.311338	0.208	0.18	0.022244	Prrc2c
Ifitm2	1.39E-06	0.376538	0.137	0.113	0.024504	Ifitm2
Cdkn1b	1.52E-06	0.355984	0.115	0.092	0.026833	Cdkn1b
H3f3a	1.60E-06	0.338344	0.239	0.213	0.02835	H3f3a
Rpl7a	1.81E-06	0.292636	0.1	0.078	0.032033	Rpl7a
Eif5b	2.23E-06	0.259868	0.18	0.153	0.039487	Eif5b
Fus	2.27E-06	0.34286	0.141	0.117	0.040203	Fus
Hmgb2	2.53E-06	0.310336	0.125	0.101	0.044672	Hmgb2

© 2023. Chen H et al. Published online at http://www.aginganddisease.org/EN/10.14336/AD.2023.0320

SUPPLEMENTARY DATA

Supplementary Table 3. Primers for Real Time RT-PCR.

Name	S/AS	Sequence	Primer(bp)	Species	Tm (°C)	Length (bp)
IFNγ	S	5’-ATGAACGCTACACACTGCATC-3’	21	mouse	55	182
	AS	5’-CCATCCTTTTGCCAGTTCCTC-3’	21			
IL-17a	S	5’-TTTAACTCCCTTGGCGCAAAA-3’	21	mouse	55	165
	AS	5’-CTTTCCCTCCGCATTGACAC-3’	20			
Irf1	S	5’-ATGCCAATCACTCGAATGCG-3’	20	mouse	55	197
	AS	5’-TTGTATCGGCCTGTGTGAATG-3’	21			
Fli1	S	5’-ATGGACGGGACTATTAAGGAGG-3’	22	mouse	55	110
	AS	5’-GAAGCAGTCATATCTGCCTTGG-3’	22			
Elf1	S	5’-TGTCCAACAGAACGACCTAGT-3’	21	mouse	55	151
	AS	5’-CACACAAGCTAGACCAGCATAA-3’	22			
Bclaf1	S	5’-CTGACCTTCGGCACGACATT-3’	20	mouse	55	184
	AS	5’-CCACCATAAGCCGTGTAAAAGAC-3’	23			
Junb	S	5’-TCACGACGACTCTTACGCAG-3’	20	mouse	55	125
	AS	5’-CCTTGAGACCCCGATAGGGA-3’	20			
Mta3	S	5’-AAGTATGGAGGTCTGAAAATGCC-3’	23	mouse	55	299
	AS	5’-AGCGTCTTACATTCTGCCCTAA-3’	22			
Ets1	S	5’-TCCTATCAGCTCGGAAGAACTC-3’	22	mouse	55	120
	AS	5’-TCTTGCTTGATGGCAAAGTAGTC-3’	23			
Elf2	S	5’-GTTCACAGCAGTAATGCTCACT-3’	22	mouse	55	85
	AS	5’-TCAAGCAGGTAGGAGATTCCAT-3’	22			
IFNγ	S	5’-TCGGTAACTGACTTGAATGTCCA-3’	23	human	55	93
	AS	5’-TCGCTTCCCTGTTTTAGCTGC-3’	21			
Irf1	S	5’-ATGCCCATCACTCGGATGC-3’	19	human	55	204
	AS	5’-CCCTGCTTTGTATCGGCCTG-3’	20			
β-actin	S	5’-GGCTGTATTCCCCTCCATCG-3’	20	mouse	55	154
	AS	5’-CCAGTTGGTAACAATGCCATGT-3’	22			
β-actin	S	5’-CATGTACGTTGCTATCCAGGC-3’	21	human	55	250
	AS	5’-CTCCTTAATGTCACGCACGAT-3’	21			

S, sense; AS, antisense, sequence; Tm, annealing temperature; length, amplicon

Supplementary Table 4. Primers for ChIP Real Time PCR.

Name	S/AS	Sequence	Primer(bp)	Species	Tm (°C)	Length (bp)
IFNγ F1	S	5’-AGTCATCCAATGTGCCAAAATAA-3’	23	human	55	155
-1670~-1516bp	AS	5’-TCTCCTTACCTGTAAAACTGGCT-3’	23			
IFNγ F2	S	5’-AGCCAGTTTTACAGGTAAGGAGA-3’	23	human	55	103
-1538~-1436bp	AS	5’-TGGGGCAAACTTGATTCCTGA-3’	21			
IFNγ F3	S	5’-TCAAGTTTGCCCCATAACTGC-3’	21	human	55	97
-1449~-1352bp	AS	5’-ATGGCACAAAAAGCCCTCCA-3’	20			
IFNγ F4	S	5’-GTGCCATCCCAAAGTGTTGTA-3’	21	human	55	201
-1359~-1159bp	AS	5’-TCAAACGCTTAATGGGCACATAC-3’	23			
IFNγ F5	S	5’-GCACCTAGCAGTATGTGCCC-3’	20	human	55	70
-1191~-1122bp	AS	5’-AAGCCTACGGTGCATCTCAA-3’	20			
IFNγ F6	S	5’-ATTGAGATGCACCGTAGGCTT-3’	21	human	55	376
-1142~-767bp	AS	5’-AGAGAATATTCCCAGGGGGAGTT-3’	23			
IFNγ F7<br>-509~-430bp	S	5’-ACTAACCAACTCTGATGAAGGACT-3’	24	human	55	80
	AS	5’-TGCAAATGACCAGAAAGCAAGG-3’	22			
IFNγ F8	S	5’-CGCATTCTTTCCTTGCTTTCTG-3’	22	human	55	73
-461~-389bp	AS	5’-TCTCAAACCTTTACAAAGGGGCA-3’	23			
IFNγ F9	S	5’-GTTCCCAACCACAAGCAAATGA-3’	22	human	55	138

© 2023. Chen H et al. Published online at http://www.aginganddisease.org/EN/10.14336/AD.2023.0320

SUPPLEMENTARY DATA

-363~-226bp	AS	5'-TGGTGGGATTCTTTGAAGGCA-3'	21			
IFNγ F10	S	5'-CAGGTGGGCATAATGGGTCT-3'	20	human	55	132
-217~-86bp	AS	5'-TGGTGACAGATAGGCAGGGAT-3'	21			
β-actin	S	5'-TGCCCAAGAGATGTCCACAC-3'	20	human	55	219
promoter	AS	5'-GAGCGAGAGCGAGATTGAGG-3'	20			
-393~-175 bp						

S, sense; AS, antisense, sequence; Tm, annealing temperature; length, amplicon

© 2023. Chen H et al. Published online at http://www.aginganddisease.org/EN/10.14336/AD.2023.0320
